# Individualized home training in head and neck cancer patients is safe and has positive short- and medium-term effects –results of a multicenter, single-arm intervention trial (OSHO #94)

**DOI:** 10.3389/fonc.2025.1602532

**Published:** 2025-06-09

**Authors:** Sabine Felser, Lars A. Bonke, Änne Glass, Daniel Strüder, Jana Stolle, Susann Schulze, Markus Blaurock, Anke Steinmetz, Julia Daunheimer, Ursula Kriesen, Christina Grosse-Thie, Christian Junghanss

**Affiliations:** ^1^ Department of Internal Medicine, Clinic III – Hematology, Oncology and Palliative Care, Rostock University Medical Center, Rostock, Germany; ^2^ Institut for Biostatistic and Informatics in Medicine, Rostock University Medical Center, Rostock, Germany; ^3^ Department of Otorhinolaryngology, Head and Neck Surgery “Otto Koerner”, Rostock University Medical Center, Rostock, Germany; ^4^ Krukenberg Cancer Center Halle, University Hospital Halle, Halle (Saale), Germany; ^5^ Department of Internal Medicine, Medical Clinic II, Carl-von-Basedow Klinikum, Merseburg, Germany; ^6^ Department of Otorhinolaryngology, Head and Neck Surgery, Universitätsmedizin Greifswald, Greifswald, Germany; ^7^ Department of Orthopedics, Trauma and Rehabilitation Medicine, Physical and Rehabilitation Medicine, University Medicine Greifswald, Greifswald, Germany; ^8^ Hematology and Oncology Practice, Rostock, Germany

**Keywords:** EORTC QLQ-C30 (version 3), EORTC QLQ-HN35, head and neck cancer, Health-related quality of life (QOL), home-based exercise, physical functionality, physical activity (exercise)

## Abstract

**Introduction:**

Despite targeted exercise interventions alleviating functional deficits in head and neck cancer (HNC) patients, many patients are insufficiently physically active. A promising approach to reducing barriers could be individually adaptable home training. The “OSHO #94” study examined the short-term effectiveness, the medium-term sustainability, and the safety of an individualized home exercise program

**Methods:**

This multicenter, single-arm, interventional study included patients in aftercare or in stable remission. Participants were advised to perform an individualized home exercise program (mobilization, coordination, strengthening, and stretching) at least three times a week and moderate-intensity endurance training two to three times a week. During the 12-week intervention, they kept a training diary and received weekly physiotherapist calls. In the subsequent 12-week follow-up (FU), participants were asked to continue training. The evaluation of short-term effects (between pre- and post-intervention) and medium-term effects (incl. FU), included the assessment of quality of life (QoL), physical activity level (Leisure Score Index (LSI); weekly amount), body composition, shoulder/cervical spine range of motion, fall risk, and aerobic performance. Adverse events were recorded.

**Results:**

Fifty-three patients (57% male) were enrolled, 83% completed the post assessment, and 72% completed FU. During the intervention, participants exercised for 257 min/week (with 95 minutes individual and 162 minutes endurance). The pre-post intervention effect on the global QoL was small (r_w_=0.20, *p*=0.186). Moderate effects were found in emotional (r_w_=0.38, *p*=0.011) and social functioning (r_w_=0.46, *p*=0.002), fatigue (r_w_=0.37, *p*=0.013), and dyspnea (r_w_=0.32, *p*=0.035). LSI increased significantly (25 *vs.* 39, *p*=0.003), whereas total physical activity duration remained unchanged (280 *vs.* 290 min/week, *p*=0.160). Small effects were observed on body composition. The largest effects were seen in physical functioning, particularly aerobic performance (r_w_=0.67, *p*<0.001). Nine participants (17%) reported training-related adverse events, primarily pain. Half of participants (48%) continued with individual training during FU. Some short-term effects could be detected medium-term.

**Discussion:**

Physical activity level improved despite an unchanged activity duration suggesting an increased training intensity. With individualized home exercises and remote support, home training was effective and safe. After support ended, patients maintained their activity level and the effects were sustained, suggesting suitability for routine care.

## 1 Introduction

Head and neck cancer (HNC) frequently lead to functional deficits, particularly in eating, breathing, speech, pain, mood, and neck and shoulder mobility (1–4). These deficits, along with visible disfigurements, weight loss, and sarcopenia (5), as well as body image disturbances (6), impacts health-related quality of life (QoL) (6–8). Advances in diagnosis and treatment have increased the survival rate of HNC patients (9–11), shifting the focus beyond life expectancy to functional abilities and QoL (12). Rehabilitative measures primarily address physical impairments (13), with physical activity emerging as a promising intervention to enhance QoL. Specifically, strength and endurance training programs have shown positive effects on muscle strength, cardiorespiratory fitness, and pain reduction (14–18). The American Head and Neck Society therefore recommends early rehabilitation screening, objective assessments, targeted referrals, and the promotion of regular exercise (19).

Despite existing knowledge and exercise recommendations, many HNC patients remain insufficiently physically active (20–22). Barriers to structured physical activity include physical complaints, time constraints, lack of motivation, and insufficient knowledge (23, 24). Moreover, individual preferences regarding the type, location, intensity, and supervision of exercise programs vary, as do limitations and needs. Surveys have shown that HNC patients prefer unsupervised and supervised training programs—performed either alone or with family members, at moderate intensity, at home or outdoors, and at flexible times (25–27). Individually adaptable home- and outdoor-based training programs are therefore considered a promising approach to promoting physical activity, yet they remain insufficiently studied. Additionally, there is limited knowledge regarding the sustainability of such (home-based) exercise interventions. Our goal was to develop a low- to moderate-intensity exercise program that HNC patients can perform independently and flexibly at home or outdoors. In the first study, suitable exercises were compiled and evaluated for their effectiveness in a group-based training setting (28). After combining suitable exercises into an exercise manual (29), the final step was to evaluate the effectiveness and safety of home-based training (30).

The primary outcome of this study was the comparison of the global QoL score after a 12-week home exercise intervention with the value measured before the intervention. The study specifically examined the suitability of the chosen approach, including its integration into routine care. To this end, (i) patients’ compliance with the training recommendations, and (ii) the perceived usefulness and actual utilization of the provided training materials and information were evaluated. Furthermore, the effectiveness of the intervention was assessed by examining (iii) the short-term effects of the home exercise program on QoL, physical activity level, body composition and physical functioning, as well as (iv) the medium-term effects beyond the intervention period. Finally, (v) the safety of the training program was assessed based on reported adverse events.

## 2 Materials and methods

### 2.1 Study design, participants, and eligibility criteria

A prospective, multicenter, single-arm, intervention trial was designed and registered (German Registry of Clinical Trials No. DRKS00023883). The Department of Hematology, Oncology, and Palliative Medicine at the University Hospital Rostock (UMR) conducted the study. Further recruiting hospitals were the Krukenberg Cancer Center Halle (UKH), and the Department of Otorhinolaryngology, Head and Neck Surgery at the University Medicine Greifswald. The study design and the number of enrolled required patients (N=53) were based on the established criteria in Felser et al. (30). Patients eligibility criteria were: ≥18 years old with a diagnosis according to ICD: C00–C14, C30–C32 (HNC) in aftercare or with stable remission under immunotherapy, who had medical clearance from their treating physician, were able to walk independently, and provided informed consent for participation. The exclusion criteria are inadequate knowledge of the German language, consent not given, clinically relevant heart failure (NYHA III and IV), myocardial infarction within the last 4 weeks, unstable angina pectoris, higher-grade valvular vitia, uncontrolled cardiac arrhythmias, chronic obstructive pulmonary disease (GOLD III and IV), peripheral arterial occlusive disease (≥Stage III according to Fontaine), diseases that could seriously impair cognitive performance (e.g. dementia, stroke, Wernicke-Korsakoff syndrome), known alcohol dependency and score <24 points on the Mini-Mental State Examination.

Elimination criteria after inclusion were diagnosis of a relapse or new tumor disease, prolonged hospital stays due to other illnesses/surgeries (e.g., heart attack, hip or knee surgery, death (=health reasons), admission to inpatient rehabilitation, and discontinuation at the patient’s request.

### 2.2 Study procedure, intervention and measurement points

Included patients underwent a comprehensive preliminary examination. Based on its results and individual goals, therapists create a personalized training plan. All patients were instructed in the training program, which included mobilization, coordination, strengthening, and stretching exercises. They received the “Exercise manual for patients with mouth, jaw, face and throat tumors’” (29) with the recommended exercises marked in it. Additionally, all recommended exercises and four comprehensive training programs were supplied as video files on a USB stick, along with an elastic resistance band and an inflatable exercise ball (Ø 22 cm) for use at home. Participants were instructed to complete either their individualized program or one of the video sessions at least three times per week for 15–30 minutes. Endurance activities (e.g., walking, cycling, swimming, dancing) were recommended 2–3 times per week for 30 minutes. Training intensity was self-monitored using the BORG rating of perceived exertion (RPE) scale (31), and the recommended range was between 11 (fairly easy) and 15 (hard). During the 12-week intervention, therapists conducted weekly telephone calls and documented the conversations. Patients were also asked to maintain a training diary. A post-examination followed after 12-week to evaluate the short-term effects (pre-post comparison).

Afterward, patients entered a 12-week follow-up (FU) period without structured support. The final FU examination was used to evaluate medium-term effects (pre-FU, post-FU) and assess whether participants maintained or enhanced outcomes independently.

### 2.3 Outcome measures

The *season* was determined based on the date of study enrollment.

*Disease-specific data* on the participants were recorded once by the treating/including physicians at the time of study inclusion.

At the three measurement points–pre, post and FU–patient-reported outcomes (PROs) were recorded, and a physical function diagnostic was carried out. The assessment consists of the following measures:

Patient-reported:

− *Sociodemographic data and lifestyle factors* were recorded using an initial questionnaire.
− *QoL* was measured using two established questionnaires: the EORTC QLQ-C30 version 3.0 questionnaire (32, 33) and the QLQ-HN35 head and neck–specific questionnaire (33). No threshold values were set in advance to identify clinically important differences. The primary outcome was determined by comparing the global QoL score post- *vs.* pre-intervention.
− *Physical activity level* were assessed using a modified version of the Godin-Shephard Leisure-Time Physical Activity Questionnaire (GSLTPAQ) (34, 35). Two parameters, the Leisure Score Index (LSI) and the weekly duration of physical activity [min], were recorded.
− *Assessment of the intervention and support* was conducted with an exit questionnaire. Participants rated the perceived effects of the intervention on ten domains (body awareness, coordination, endurance, general mood, general performance, mobility, physical well-being, self-esteem, strength, and stress reduction) using a 4-point Likert scale. They also evaluated the difficulty of self-motivation and the motivational impact of the weekly physiotherapy calls using the same scale. Patients indicated whether they exercised alone or with others, whether they intended to continue training, and whether the provided information was helpful. Finally, they were asked to which material they had used (exercise manual, videos, and training equipment).
− During the FU examination, patients were asked about the *continuation of the individual exercise program* even after the end of the intervention.

Physical function diagnostic:

− B*ody composition*, including body fat percentage [%], and skeletal muscle mass [kg] was assessed using a bioimpedance analyzer (BIA). These measures were optional, as not all recruiting clinics had access to a BIA. UMR and UKH used the same device, the ‘seca mBCA 525’ (seca GmbH, Hamburg, Germany). Measurements were conducted according to the manufacturer’s instructions. The body mass index [kg/m²] was calculated based on height and weight.
− The *mobility of the temporomandibular joints* was measured using the distance between the incisors [cm] determined with a ruler at maximum mouth opening.
− *Shoulder joint mobility* was measured as the active range of motion (ROM) in the sagittal, frontal, and transversal planes using a manual goniometer [°]. Patients and therapists indicated separately for each shoulder joint whether there were restrictions in at least one axis of motion.
− *Cervical* sp*ine mobility* was measured as the active ROM in rotation and lateral flexion using a manual goniometer [°] and as inclination/reclination using a ruler [cm].
− The *mobility of the lower back and hamstring muscles* was assessed with the stand and reach test [cm] (36).
− *Fall risk* was evaluated by using the short physical performance battery (SPPB) (37). The scores range from 0 (worst performance) to 12 (best performance).
− *Aerobic performance* was assessed using the 6-minute walk test (6MWT) (38). The primary measure was the walking distance [m]. After the 6MWT, participants rated their RPE using the Borg scale (6 = really, really easy, 20 = maximum effort) (31) and their exercise-induced pain in the leg muscles using a Category-Ratio (CR)-10 scale (0 = no pain at all, 10 = extremely intense pain) (39).

Following completion of the intervention, the telephone protocols and training diaries were evaluated regarding the following parameters:

− *Compliance regarding training recommendations*: training diary were analyzed for average weekly training frequency and duration, separately for individualized and endurance training. Additional, non- recommendation activities (e.g., yoga) were recorded separately.
− *Adverse events* in connection with the training were recorded.
− The *reason for dropping out* was asked in the event of early termination of the study, if possible.

### 2.4 Statistical analysis

Quantitative variables are presented as mean ± standard deviation (SD) or in case of non-normality, as median (Q1, Q3), ranging from minimum to maximum (min to max). Qualitative variables are reported as relative frequency % and absolute (n). Missing data are indicated but excluded from percentage calculations. The normality of the data was assessed using the Shapiro-Wilk test.

Spearman’s rank correlation was performed to determine whether there is a correlation between intrinsic and extrinsic motivation (weekly phone calls).

Pre-post analyses were conducted using the paired t-test for normally distributed data with Cohen’s d for dependent samples to estimate short-term effects of intervention (0.20 to <0.5 = small, 0.5 to <0.8 = moderate, ≥0.80 = large) (40). Otherwise, the Wilcoxon signed-rank test with r_w_ estimating the effect was used. Effect sizes r_w_ were assessed on the basis of Cohen’s thresholds (0.10 to <0.3 = small, 0.3 to 0.5 = moderate, ≥0.5 = large) (41).

Medium-term effects of intervention were examined as changes between pre- and FU measurement, as well as between post- and FU measurement. To avoid attrition bias, only participants who completed the whole study (all three measurement points) were included into the analysis of medium-term effects. Thus, the two different samples analyzed pre-post (n=44) and pre-(post)-FU (remained n=38) could cause differing pre-post results. For normally distributed quantitative data, a one-way repeated-measures ANOVA with Bonferroni correction was conducted. The assumption of normality of the difference scores was assessed using the Shapiro-Wilk test. No outliers were detected in the data. Cohen’s d is given to quantify effects. For non-normally distributed data, a Friedman test was conducted, followed by Wilcoxon *post hoc* tests with a Bonferroni correction. Box plots were used to graphically represent changes over time (**Supplementary Figures S3**, **S4**). A *p*-value <0.05 was considered statistically significant. All data were analyzed using IBM^®^ SPSS^®^ Statistics.

## 3 Results

**Figure 1:**
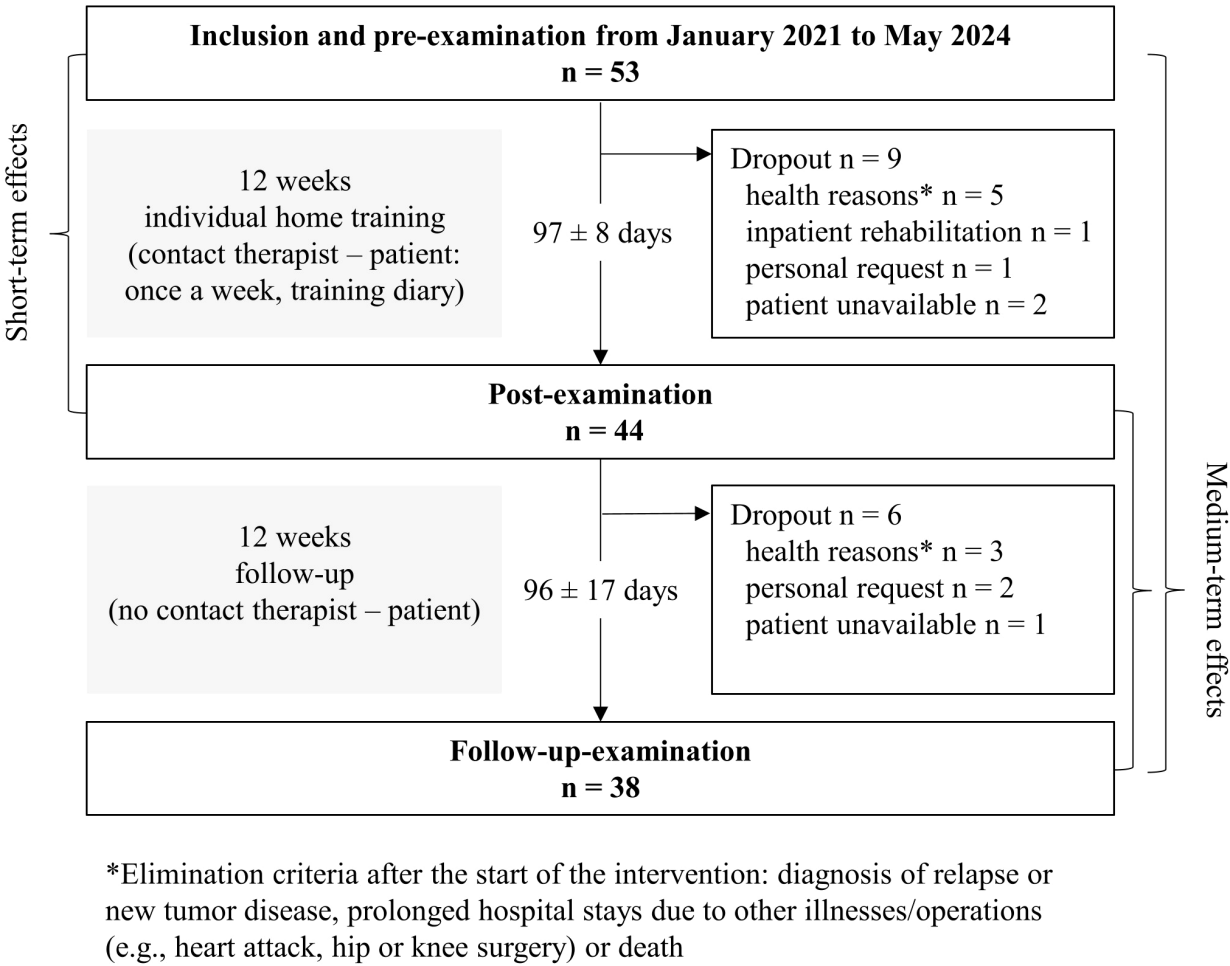
Flow chart of the OSHO #94 study.

Fifty-three patients were enrolled in the study between January 2021 and May 2024. The 12-week home exercise program and post-examination were completed by 83% (n=44). The final FU examination could be carried out on 72% (n=38) of the participants. A total of 28% (n=15) of participants discontinued the study prematurely. Of these, eight withdrew for health reasons, one due to inpatient rehabilitation, and three for personal reasons. Three patients could not be reached by phone, leaving the reasons for their discontinuation unclear (**Figure 1**).

Most participants were enrolled in the study during spring (40%, n=21) and fall (30%, n=16). In winter, 24% (n=13) were included, while 6% (n=3) were enrolled in summer.

### 3.1 Participants’ sociodemographic, lifestyle and clinical data

The participants’ sociodemographic, lifestyle, and clinical data are summarized in **Table 1**. Of the 53 participants included, 57% (n=30) were male. The median age was 63 (58, 70), ranging from 20 to 85 years. An upper secondary education had been attained by 38% (n=20) of the participants, 58% (n=30) were non-smokers, 35% (n=18) answered that they currently do not drink any alcohol at all, and 60% (n=31) stated that they had been active in sports before the disease. The most common tumor location was the oropharynx (36%, n=19), followed by the oral cavity (26%, n=14). The median time after the (last) diagnosis was 28 (12, 51) months (3 to 199). At the time of study enrollment, 91% (n=48) were in complete remission. A feeding tube was present in 13% (n=7) of the participants and 38% (n=20) were taking pain medication.

**Table 1:** Sociodemographic, lifestyle and clinical data (n=53).

Patients characteristics	Category	Values n (%) or median (Q1, Q3)
Gender	Women Men	23 (43) 30 (57)
Age [years]		63 (58, 70)
School education [years]	Lower secondary education Upper secondary education	33 (62) 20 (38)
Family status	Living alone Married/living with a partner	18 (34) 35 (66)
Professional status	Working Retired Other	14 (26) 35 (66) 4 (8)
Tobacco consumption	Smoker Ex-Smoker Non-Smoker	6 (11) 16 (31) 30 (58)
Current alcohol consumption	Yes No	34 (65) 18 (35)
Active in sports before cancer diagnosis	Yes No	31 (60) 21 (40)
Tumor location	Oropharynx Oral cavity Others	19 (36) 14 (26) 20 (38)
UICC-cancer stage	I II III IV	16 (31) 7 (13) 11 (21) 18 (35)
Time after initial diagnosis [months]		28 (12, 51)
Current therapy situation	Complete remission Under immunotherapy Others	48 (91) 1 (2) 4 (7)
Treatment	Surgery only RT or RCT only Surgery and RT/RCT	8 (15) 10 (19) 35 (66)
Feeding tube present at baseline	Yes No	7 (13) 46 (87)
Taking nutritional supplements at baseline	Yes No	19 (36) 34 (64)
Taking painkillers at baseline	Yes No	20 (38) 33 (62)

Data are presented as the number of participants (%) for categorical variables and as median (Q1, Q3) for continuous variables.

RT, radiotherapy; RCT, combined radio-chemotherapy; UICC, Union for International Cancer Control.

### 3.2 Compliance regarding training recommendations

The training diaries of n=41 participants were available with one of them only partially analyzable, finally resulting in n=40 valid training records. Individual exercises were completed 3.3 (2.2, 4.3) times per week, ranging from 0.0 to 6.8, with a training time of 95 (56, 127) min (0 to 256). Endurance training, often in the form of walking, was completed 2.7 (1.6, 5.5) times per week (0 to 13.8), with 162 (86, 304) min (0 to 531 min). Additional, non- recommendation activities was only completed to a limited extent. The weekly training overall was completed 6.4 (4.9, 9.2) times, with a median training time of 257 (162, 417) min, ranging from 39 to 753 min.

Overall, 85% (35/41) of the participants followed the recommendations regarding their individual exercise program with a given number of sessions to practice, and a little less, 78% (31/40) regarding their endurance training. The proportion of patients who met the training recommendations per the protocol in both areas was 68% (28/40). A comparison of training recommendations and realizations by the participants is shown in **Supplementary Figure S1**.

According to training diaries and telephone protocols, 58% (25/43) of participants experienced training interruptions. The longest (4 weeks) followed a feeding tube placement. COVID-19 and colds led to 4–14-day interruptions in five cases, while pneumonia (n=1) and urinary tract infections (n=2) caused 1–2-week breaks. Five participants paused their training during the end-of-year celebrations. Shorter interruptions (1–4 days) were due to pain (migraine/headache, back pain, abdominal pain, menstrual pain), dizziness, nausea, medical appointments, vaccinations, personal stress, childcare, travel, or weather conditions. Depression and lack of motivation led to up to 14-day breaks in three cases.

During the post-examination, 67% (n=28) said “yes” to the question of whether they would continue training, while 33% (n=14) “I don’t know.” At FU, 48% had maintained individual training.

### 3.3 Information, use of the exercise materials, weekly calls, and training with others

All patients (100%) stated that the information they received about the training was sufficient. The exercise manual was used by 81% (n=34) of the participants and the USB stick with the training videos by 41% (n=17), with 21% (n=9) stating that they used both. Small equipment (elastic band/ball) was used by 83% (n=35) of the participants. During the post-examination, 23% (n=10) found self-motivation for training not difficult, 40% (n=17) somewhat difficult, 30% (n=13) moderately difficult, and 16% (n=7) very difficult. Overall, 93% (n=40) felt that the therapists’ weekly calls positively influenced their motivation (26% very, 37% quite, 26% somewhat). No correlation was found between intrinsic and extrinsic motivation. Most participants (75%, n=32) trained alone, 9% (n=4) with others, and 16% (n=7) both alone and with others.

### 3.4 Moderate short-term effects on QoL

The short-term intervention effects on QoL, measured pre-post, are reported in **Table 2**.

**Table 2:** Moderate pre-post-intervention effects on quality of life and physical activity level (n=44).

Parameter	Pre Median (Q1, Q3)	Post Median (Q1, Q3)	Effect size r_w_	*p-*value
QoL (EORTC QLQ-C30^a^)				
Global QoL scale [0-100]	63 (50, 75)	67 (58, 75)	0.20	0.186
Functional scales [0-100]				
physical role emotional cognitive social	83 (73, 92) 67 (50, 100) 63 (42, 83) 75 (50, 96) 67 (50, 83)	87 (73, 93) 67 (67, 100) 75 (58, 92) 75 (67, 100) 83 (54, 100)	0.23 0.12 **0.38** 0.28 **0.46**	0.123 0.437 0.011 0.059 0.002
Symptom scales [0-100]				
fatigue nausea/vomiting pain	33 (22, 64) 0 (0, 0) 33 (0, 63)	33 (11, 56) 0 (0, 0) 25 (0, 63)	**0.37** 0.05 0.15	0.013 0.719 0.324
Single items [0-100]				
dyspnea insomnia appetite loss constipation diarrhea financial difficulties	33 (0, 33) 33 (0, 67) 17 (0, 33) 0 (0, 33) 0 (0, 0) 33 (0, 58)	0 (0, 33) 33 (0, 67) 0 (0, 58) 0 (0, 33) 0 (0, 0) 0 (0, 33)	**0.32** 0.08 0.10 0.21 0.19 0.21	0.035 0.618 0.517 0.162 0.198 0.066
QoL (EOTRC QLQ-HN35^b^)				
Symptom scales [0-100]				
pain swallowing taste/smell speech social eating social contacts sexuality	25 (10, 42) 17 (8, 46) 33 (0, 50) 22 (11, 44) 25 (8, 58) 13 (0, 33) 33 (0, 67)	17 (2, 42) 17 (8, 42) 17 (0, 33) 22 (0, 30) 17 (8, 50) 7 (0, 25) 17 (0, 33)	0.26 0.15 **0.34** **0.36** **0.30** 0.26 **0.49**	0.083 0.312 0.023 0.016 0.045 0.058 0.001
Symptom items [0-100]				
teeth problems trismus dry mouth sticky saliva cough feeling ill	33 (0, 67) 33 (0, 67) 67 (42, 100) 33 (33, 67) 33 (33, 67) 17 (0, 33)	0 (0, 33) 33 (0, 67) 67 (33, 100) 33 (0, 67) 33 (0, 33) 0 (0, 33)	0.28 0.16 **0.47** 0.22 0.20 0.23	0.065 0.292 0.002 0.133 0.179 0.134
Physical activity (GSLTPAQ^c^)				
Leisure Score Index [≥0] duration [min per week]	25 (20, 49) 280 (170, 555)	39 (28, 64) 290 (195, 566)	**0.46** 0.22	0.003 0.160

The Wilcoxon signed-rank test was used to compare pre and post scores.

Interpretation of effect size r_w_ 0.1 to <0.3 = small, 0.3 to <0.5 = moderate, ≥0.5 large effect.

bold: moderate intervention effects of statistical significance.

QoL, Quality of Life.

a Quality of Life questionnaire of cancer patients of European Organization for Research and Treatment of Cancer.

b Head and neck–specific questionnaire of European Organization for Research and Treatment of Cancer.

A high value on the scale ‘global QoL’ and on the functional scales means a high degree of subjectively perceived health and a high assessment of the QoL or a high degree of performance and function. A high value in the symptom scales correlates with a high degree of complaints.

c GSLTPAQ, Godin-Shepard Leisure-Time Physical Activity Questionnaire.

*EORTC QLQ-C30:* The intervention effect on the global QoL was small (r_w_=0.20, *p*=0.186). The score improved from 63 (pre intervention) to 67 (post intervention) by 4 points. Moderate effects were observed in 2 of the 5 functional scales: emotional functioning (r_w_=0.38, *p=0.011*, +12 points) and social functioning (r_w_=0.46, *p=0.002*, +16 points); the 3 remaining effects were detected as small with r_w_<0.3. Among the 3 symptom scales and 6 single items, moderate positive effects were observed for fatigue (r_w_=0.37*, p*=0.013) and dyspnea (r_w_=0.32, *p*=0.035).

*EORTC QLQ-HN35:* Positive changes were observed in all 7 symptom scales, with significant effects for taste/smell (r_w_=0.34, *p*=0.023), speech (r_w_=0.36*, p*=0.016), social eating (r_w_=0.30*, p*=0.045), and sexuality (r_w_=0.49*, p*=0.001). A significant and moderate effect was observed for dry mouth (r_w_=0.47*, p*=0.002), while the effects for the other 5 symptom items were small.

### 3.5 Short-term effects on physical activity level

The home training intervention had a moderate effect on the physical activity level, with the LSI increasing from 25 to 39 (r_w_=0.46, *p*=0.003). By contrast, the effect on weekly physical activity duration was not significant (r_w_=0.22, *p*=0.160, 280 *vs.* 290 minutes per week) (**Table 2**).

### 3.6 Short-term effects on body composition

The intervention had small favorable effects on body composition (**Table 3**). Between the pre- and post-intervention measurements, body fat percentage decreased by 1.2%p (d=0.48, *p*=0.009, n=33) and skeletal muscle mass increased by about 3%p (d=0.45, *p*=0.014, n=33). No significant changes were observed in body mass index (r_w_=0.03, *p*=0.848, n=39).

**Table 3:** Moderate and large pre-post-intervention effects on body composition and physical functionality.

Parameter	n	Pre	Post	Effect size d	Effect size r_w_	*p*-value
Body composition						
Body Mass Index [kg/m²] Body fat [%] Skeletal muscle mass [kg]	39 33 33	23.8 (20.8, 26.6) 27.8 ± 8.2 23.9 ± 7.4	23.7 (21.0, 26.6) 26.6 ± 8.2 24.6 ± 7.4	**0.48** **0.45**	0.03	0.848 0.009 0.014
Physical functional status						
Mobility						
Inter-incisor distance [cm]	30	4.0 (3.4, 4.4)	4.3 (3.5, 4.6)		**0.61**	<0.001
ROM shoulder joint [°] - only patients with restricted mobility						
ante-retro version right ante-retro version left abduction-adduction right abduction-adduction left internal-external rotation right internal-external rotation left	16 16 17 15 18 14	177 ± 39 169 (160, 186) 123 (120, 130) 119 ± 19 116 (99, 130) 118 (97, 128)	189 ± 38 178 (162, 186) 130 (128, 137) 129 ± 8 129 (120, 132) 124 (108, 133)	**0.77** **0.62**	0.37 **0.69** **0.47** 0.32	0.002 0.139 0.004 0.017 0.047 0.235
ROM cervical spine – only patients with restricted mobility						
Inclination-reclination [cm] lateral flexion [°] rotation [°]	34 32 34	13.7 ± 3.7 45 (35, 54) 96.4 ± 26.7	14.2 ± 3.6 59 (42, 69) 104.2 ± 25.4	0.21 0.32	**0.44**	0.238 0.012 0.069
Stand and reach test [cm]	42	-8.4 ± 12.0	-6.4 ± 11.0	**0.32**		0.046
Fall risk						
SPPB [score 0 to 12]	42	11.5 (10.0, 12.0)	12.0 (11.0, 12.0)		**0.35**	0.023
Aerobic performance						
6-min walk distance [m] Rating of perceived exertion (Borg scale) Exercise-induced leg pain (CR-10 scale)	38 38 38	551 (500, 589) 12.5 (11.0, 13.0) 0.5 (0.0, 2.0)	582 (531, 666) 12.5 (11.0, 14.0) 0.5 (0.0, 2.1)		**0.67** 0.10 0.04	<0.001 0.534 0.793

Variables are presented as mean ± standard deviation or, in case of non-normality, as median (Q1, Q3).

The paired t test or in case of non-normality, Wilcoxon test was used to compare pre and post scores.

Interpretation of effect size Cohen’s d for paired t test 0.20 to <0.5 = small, 0.5 to <0.8 = moderate, ≥0.80 = large effect.

Interpretation of effect size r_w_ (Wilcoxon test) 0.1 to <0.3 = small, 0.3 to <0.5 = moderate, ≥0.5 large effect.

bold: significant moderate and large intervention effects.

ROM, range of motion; SPPB, short physical performance battery; CR, Category-Ratio.

### 3.7 Short-term effects on physical functioning

*Inter-incisor distance:* Mouth opening increased by 3 mm post-intervention (r_w_=0.61, *p*< 0.001, n=30), see **Table 3**.

*ROM in the shoulder joints:* In the ROM of the shoulder joints, moderate to large effects were observed in patients with restricted mobility. Thus, the ROM in the right shoulder joint improved in ante-/retroversion by 12° (d=0.77, *p*=0.002, n=16), in abduction/adduction by 7° (r_w_=0.69, *p*=0.004, n=17), and in internal/external rotation by 13° (r_w_=0.47, *p*=0.047, n=18). On the left side, an improvement of 10° (r_w_=0.62, *p*=0.017, n=16) was observed in abduction/adduction. No statistically significant effects could be detected in the ROM of the left shoulder joint for ante-/retroversion and internal/external rotation, since the changes here were rather small.

*ROM in the cervical* sp*ine:* The intervention had a moderate effect on lateral flexion in patients with limited cervical spine mobility: +14° (r_w_=0.44, *p*=0.012, n=32). The effects on rotation were small: +8° (d=0.32, *p*=0.069, n=34). No statistically significant changes were observed in inclination/reclination.

*Stand and reach test:* The intervention effect on mobility of the lower back and hamstring was small (d=0.32, *p*=0.046, n=42). The distance to the ground decreased by 2.0 cm after the intervention.

*SPPB:* Before the intervention, 50% of participants achieved the maximum score, increasing to 67% post-intervention. The calculated effect was small (r_w_=0.35, *p*=0.023, n=42).

*Aerobic performance (6MWT):* A large intervention effect on aerobic performance was observed (r_w_=0.67, *p*<0.01, n=38): participants increased their walking distance by an average of 31 m (+5.6%) post-intervention. No statistically significant changes were observed in RPE (BORG scale) or exercise-induced leg muscle pain (CR-10 scale).

*PROs:* The subjective rating of the improvements by the participants (none = 0 to very strong = 3) shows similar distributions across all 10 domains surveyed. Mean values ranged between 1.67±0.75 and 1.40±0.66 (slightly to fairly), with the highest values given for mobility, body awareness and coordination, and the lowest for endurance and strength (**Supplementary Figure S2**).

### 3.8 Small medium-term effects on QoL

Medium-term intervention effects on QoL, comparing both, pre-FU and post-FU, are reported in **Table 4**, with additionally given the pre-post results of n=38 permanent participants. All changes of scores are shown graphically as box plots in **Supplementary Figure S3**.

**Table 4:** Small medium-term intervention effects (pre-FU, post-FU) on quality of life and physical activity levels (n=38).

Parameter	Pre median (Q1, Q3)	Post median (Q1, Q3)	FU median (Q1, Q3)	Pre-Post 12 weeks effect size r_w_ (*p*)	Post-FU 12 weeks effect size r_w_ (*p*)	Pre-FU 24 weeks effect size r_w_ (*p*)
QoL (EORTC QLQ-C30^a^)						
Global QoL scale [0-100]	67 (50, 75)	67 (58, 83)	75 (65, 83)	0.04 (0.685)	0.03 (1.000)	0.07 (0.175)
Functional scales [0-100]						
physical role emotional cognitive social	87 (78, 93) 67 (63, 100) 63 (42, 83) 83 (63, 100) 67 (50, 83)	87 (80, 93) 67 (67, 100) 75 (58, 92) 83 (67, 100) 83 (63, 100)	87 (73, 93) 75 (63, 100) 75 (58, 85) 83 (67, 83) 83 (67, 100)	0.04 (0.906) 0.01 (1.000) 0.08 (0.117) 0.04 (0.828) 0.09 (0.041)	0.01 (1.000) 0.01 (1.000) 0.00 (1.000) 0.02 (1.000) 0.00 (1.000)	0.04 (0.685) 0.00 (1.000) 0.08 (0.076) 0.02 (1.000) 0.09 (0.056)
Symptom scales [0-100]						
fatigue nausea/vomiting pain	33 (22, 56) 0 (0, 0) 33 (0, 50)	33 (11, 44) 0 (0, 0) 17 (0, 54)	33 (11, 44) 0 (0, 0) 25 (0, 33)	0.08 (0.101) 0.01 (1.000) 0.01 (1.000)	0.00 (1.000) 0.00 (1.000) 0.05 (0.506)	0.06 (0.408) 0.01 (1.000) 0.06 (0.256)
Single items [0-100]						
dyspnea insomnia appetite loss constipation diarrhea financial difficulties	33 (0, 33) 33 (0, 67) 0 (0, 33) 0 (0, 33) 0 (0, 0) 33 (0, 42)	0 (0, 33) 33 (0, 67) 0 (0, 41) 0 (0, 33) 0 (0, 0) 0 (0, 33)	0 (0, 33) 33 (0, 67) 0 (0, 33) 0 (0, 33) 0 (0, 0) 0 (0, 33)	0.05 (0.561) 0.01 (1.000) 0.03 (1.000) 0.02 (1.000) 0.01 (1.000) 0.10 (0.030)	0.02 (1.000) 0.03 (1.000) 0.01 (1.000) 0.01 (1.000) 0.00 (1.000) 0.00 (1.000)	0.03 (1.000) 0.02 (1.000) 0.03 (1.000) 0.00 (1.000) 0.01 (1.000) **0.10 (0.030)**
QoL (EOTRC QLQ-HN35^b^)						
Symptom scales [0-100]						
pain swallowing taste/smell speech social eating social contacts sexuality	25 (8, 42) 17 (8, 33) 33 (0, 50) 22 (11, 44) 25 (0, 50) 10 (0, 33) 33 (17, 67)	17 (0, 42) 8 (0, 35) 17 (0, 33) 22 (0, 44) 17 (0, 44) 7 (0, 22) 17 (0, 33)	17 (0, 33) 17 (0, 25) 8 (0, 33) 11 (0, 36) 13 (0, 35) 7 (0, 27) 33 (0, 50)	0.07 (0.175) 0.03 (1.000) 0.04 (0.685) 0.06 (0.256) 0.04 (0.906) 0.05 (0.506) 0.10 (0.025)	0.05 (0.506) 0.04 (0.989) 0.03 (1.000) 0.03 (1.000) 0.04 (0.906) 0.01 (1.000) 0.04 (0.754)	**0.12 (0.003)** 0.07 (0.226) 0.07 (0.175) 0.09 (0.048) 0.08 (0.117) 0.03 (0.906) 0.06 (0.408)
Symptom items [0-100]						
teeth problems trismus dry mouth sticky saliva cough feeling ill	33 (0, 67) 33 (0, 75) 67 (33, 100) 33 (0, 67) 33 (25, 67) 0 (0, 33)	0 (0, 33) 33 (0, 67) 67 (33, 75) 33 (0, 67) 33 (0, 33) 0 (0, 33)	33 (0, 42) 0 (0, 33) 33 (33, 67) 33 (0, 67) 33 (0, 33) 0 (0, 33)	0.08 (0.117) 0.06 (0.408) 0.09 (0.056) 0.07 (0.226) 0.03 (1.000) 0.04 (0.989)	0.07 (0.175) 0.04 (0.754) 0.02 (1.000) 0.01 (1.000) 0.01 (1.000) 0.01 (1.000)	0.01 (1.000) **0.10 (0.025)** **0.10 (0.015)** 0.07 (0.134) 0.04 (0.828) 0.05 (0.621)
Physical activity (GSLTPAQ^c^)						
Leisure Score Index [≥0] duration [min per week]	27 (21, 52) 280 (160, 570)	38 (27, 60) 290 (199, 551)	43 (25, 60) 325 (210, 518)	0.09 (0.065) 0.06 (0.361)	0.01 (1.000) 0.02 (1.000)	0.08 (0.155) 0.05 (0.769)

A Friedman test was performed, followed by *post hoc* Wilcoxon tests with Bonferroni correction was conducted to analyze temporal changes within the sample.

Interpretation of effect size r_w_ 0.1 to <0.3 = small, 0.3 to <0.5 = moderate, ≥0.5 large effect.

bold: significant small medium-terms intervention effects.

QoL, Quality of Life; FU, follow-up.

a Quality of Life questionnaire of cancer patients of European Organization for Research and Treatment of Cancer.

b Head and neck–specific questionnaire of European Organization for Research and Treatment of Cancer.

A high value on the scale ‘global QoL’ and on the functional scales means a high degree of subjectively perceived health and a high assessment of the QoL or a high degree of performance and function. A high value in the symptom scales correlates with a high degree of complaints.

c GSLTPAQ, Godin-Shepard Leisure-Time Physical Activity Questionnaire.

*EORTC QLQ-C30:* No significant effect on global QoL was observed between pre and FU examination (r_w_=0.07, *p*=0.175, n=38), though the score itself changed by +8 points. But note, this increase is only a descriptive information and reported not before FU (i.e. not by intervention), namely from the post intervention situation with a score of 67 to 75 FU. The resulting effect on QoL (post-FU) is not significant (r_w_=0.03, *p*=1.000). Similarly, no significant changes were observed post-FU in both, the functional scales, symptom scales and single items.

*EORTC QLQ-HN35*: Positive changes with small effects between pre and FU measurement were observed in 1 of the 7 symptom scales: pain (r_w_=0.12, *p*=0.003), as well as in 2 of the 6 individual items: trismus (r_w_=0.10, *p*=0.025), and dry mouth (r_w_=0.10, *p*=0.015).

### 3.9 Medium-term effects on physical activity level

The LSI and the weekly training duration continue to increase, but there are no significant effects (**Table 4**).

### 3.10 Medium-term effects on body composition

The small positive effects on body composition seen in short-term analysis are not detectable between post and FU. The score values are similar. Between pre intervention and FU, body fat decreased by 1.1%p (d=0.43, *p*=0.086, n=29) and muscle mass increased by about 3%p (d=0.36, *p*=0.189, n=29), which is consistent with the short-term results (**Table 5**).

**Table 5:** Small and moderate medium-term intervention effects (pre-FU, post-FU) on body composition and physical functionality.

Parameter	n	Pre	Post	FU	Pre-post 12 weeks effect size (*p*)	Post-FU 12 weeks effect size (*p*)	Pre-FU 24 weeks effect size (*p*)
Body composition							
Body Mass Index [kg/m²] Body fat [%] Skeletal muscle mass [kg]	31 29 29	23.8 (20.8, 27.1) 28.4 ± 8.2 23.9 ± 7.4	23.8 (21.0, 26.6) 27.4 ± 8.1 24.7 ± 7.5	23.5 (20.8, 26.4) 27.3 ± 9.4 24.6 ± 7.1	r_w_=0.00 (1.000) d=0.46 (0.060) d=0.44 (0.077)	r_w_=0.03 (1.000) d=0.07 (1.000) d=0.09 (1.000)	r_w_=0.03 (1.000) d=0.43 (0.086) d=0.36 (0.189)
Physical functional status							
Mobility							
Inter-incisor distance [cm]	27	4.0 (3.4, 4.4)	4.3 (3.5, 4.6)	4.2 (3.5, 5.0)	r_w_=0.10 (0.145)	r_w_=0.01 (1.000)	r_w_=0.11 (0.105)
ROM shoulder joint [°] - *only patients with restricted mobility*							
ante-retro version right ante-retro version left abduction-adduction right abduction-adduction left internal-external rotation right internal-external rotation left	13 14 14 13 15 11	180 (165, 199) 170 (159, 189) 127 (120, 131) 127 (113, 130) 120 (110, 130) 115 (99, 130)	194 (173, 213) 180 (168, 189) 130 (129, 137) 130 (126, 137) 128 (118, 133) 128 (108, 132)	194 (170, 211) 184 (167, 190) 130 (125, 134) 130 (126, 131) 128 (118, 132) 128 (110, 130)	r_w_=0.22 (0.039) r_w_=0.19 (0.176) r_w_=0.27 (0.008) r_w_=0.17 (0.359) r_w_=0.17 (0.204) r_w_=0.22 (0.264)	r_w_=0.00 (1.000) r_w_=0.02 (1.000) r_w_=0.08 (0.450) r_w_=0.09 (1.000) r_w_=0.09 (1.000) r_w_=0.11 (1.000)	**r_w_=0.22 (0.039)** r_w_=0.21 (0.113) r_w_=0.19 (0.059) r_w_=0.09 (1.000) r_w_=0.09 (1.000) r_w_=0.11 (1.000)
ROM cervical spine – *only patients with restricted mobility*							
Inclination-reclination [cm] lateral flexion [°] rotation [°]	30 28 30	14.3 ± 3.4 45 (35, 54) 97.3 ± 26.8	14.7 ± 3.5 59 (39, 69) 104.8 ± 26.2	14.4 ± 2.8 60 (32, 70) 104.3 ± 23.1	d=0.17 (1.000) r_w_=0.08 (0.373) d=0.30 (0.335)	d=0.16 (1.000) r_w_=0.03 (1.000) d= 0.04 (1.000)	d=0.05 (1.000) r_w_=0.04 (1.000) d=0.28 (0.407)
Stand and reach test [cm]	34	-6.3 ± 10.6	-4.8 ± 10.3	-2.8 ± 11.0	d=0.27 (0.387)	d=0.32 (0.212)	**d=0.53 (0.012)**
Fall risk							
SPPB [score]	35	12.0 (10.0, 12.0)	12.0 (11.0, 12.0)	12.0 (11.0, 12.0)	r_w_=0.07 (0.249)	r_w_=0.00 (1.000)	r_w_=0.07 (0.192)
Aerobic performance							
6-min walk distance [m] Rating of perceived exertion (Borg scale) Exercise-induced leg pain (CR-10 scale)	31	554 (500; 570) 13.0 (11.0; 13.0) 0.5 (0.0; 2.0)	585 (537; 665) 12.0 (11.0; 14.0) 0.5 (0.0; 1.5)	584 (520; 643) 12.0 (9.0; 13.0) 0.0 (0.0; 1.0)	r_w_=0.13 (0.010) r_w_=0.02 (1.000) r_w_=0.01 (1.000)	r_w_=0.00 (1.000) r_w_=0.03 (1.000) r_w_=0.05 (0.841)	**r_w_=0.14 (0.009)** r_w_=0.01 (1.000) r_w_=0.05 (0.683)

Variables are presented as mean ± standard deviation or in case of non-normality, as median (Q1, Q3).

A one-way repeated measures ANOVA, Bonferroni-corrected pairwise t-tests or in case of non-normality, a Friedman test including *post hoc* Wilcoxon tests with Bonferroni correction was used to compare the values between the three measurement points (pre, post, follow-up (FU)).

Interpretation of effect size Cohen’s d for paired t test 0.20 to <0.5 = small, 0.5 to <0.8 = moderate, ≥0.80 = large effect.

Interpretation of effect size r_w_ (Wilcoxon test) 0.1 to <0.3 = small, 0.3 to <0.5 = moderate, ≥0.5 large effect.

bold: significant small and moderate medium-terms intervention effects.

ROM, range of motion; SPPB, short physical performance battery; CR, Category-Ratio.

### 3.11 Medium-term effects on physical functioning

The medium-term intervention effects on physical functioning are reported in **Table 5**, and graphically as box plots in **Supplementary Figure S4**.

The score values concerning FU *vs.* post intervention are comparable. No significant effects were observed between post- and FU examination. In contrast, the shown short-term increase of +2 cm mobility in the stand and reach test increased further during FU for two more cm, so that a moderate effect was observed pre-FU (d=0.53, *p=0.012*, n=34). Small significant effects pre-FU are observed in patients with restricted mobility in the shoulder joint in the right ante-retroversion (r_w_=0.22, *p*=0.039, n=13) and in aerobic performance assessed with the 6MWT (r_w_=0.14, *p*=0.009, n=31).

### 3.12 Safety

A total of nine participants (17%) reported adverse events that could be related to exercise. One participant each reported brief dizziness after head movements, nausea after training, cramps during activity in the shoulder girdle/forearm/thumb, Achilles tendon problems, pain during single-leg stance, knee pain, neck pain, and muscle pain. None of these events led to hospitalizations, long-term impairments, or damage.

## 4 Discussion

The three key findings of our study are: First, a low- to moderate-intensity individualized home exercise program with remote support is safe for HNC patients in the aftercare; second, positive effects on QoL, physical activity level, body composition and physical function were observed, and third, HNC patients accept and use the training materials and knowledge provided.

### 4.1 Recruitment and participants

In this multicenter study conducted in Rostock, Halle and Greifswald, 53 participants were recruited over a period of 3.5 years. The long recruitment period, aside from the restrictions imposed by the COVID-19 pandemic, may be attributed to the limited number of participating centers (three university hospitals). In Germany, oncological exercise therapy is not yet widely implemented in clinical settings, resulting in a shortage of exercise therapists available to support patients. Recruitment was carried out during aftercare appointments; via self-help group; and through flyers, posters, and ear, nose, and throat practices. Due to the broad and non-standardized nature of these recruitment strategies, it is difficult to estimate the recruitment rate. Seasonal variations were also observed, with spring and fall appearing to the most favorable periods for engaging HNC patients in physical activity programs. The proportion of women (43%) was notably higher than the typical HNC gender distribution (90% men, 10% women). Additionally, many participants were non-smokers and had prior physical activity experience, indicating a selection bias and limiting the generalizability of the findings to the broader HNC population in Germany.

### 4.2 Compliance with training recommendations

The individual home exercise program is a novel set of exercises specifically designed for HNC patients in aftercare (42). One objective of this study was to assess participants’ training frequency and amount per week. Unlike the pilot cohort (28), participants were explicitly encouraged to incorporate additional endurance training in line with cancer survivor guidelines (43) and the recommendations for HNC patients (16).

Our findings showed that two-thirds of participants adhered to the exercise recommendations. The realized training corresponded to training recommendations in either terms of weekly training session or total duration, or even both, with unexpectedly high compliance (85%) to individual training. This adherence was comparable to that in other home-based (44) and group-based studies (45–47) and significantly higher than that in HNC patients undergoing chemotherapy (48). While the number of weekly sessions met expectations, total training volume was twice the target, despite intermittent interruptions in over 50% of participants. This may be attributed to high intrinsic motivation and good physical performance (selection bias). Most participants were already physically active before the intervention (LSI ≥24) (35). Since the training duration remained stable as the LSI increased, it can be inferred that participants intensified their workouts, which corresponds to training progression (49).

### 4.3 Information, use of the exercise materials, weekly calls, and training with others

The study aimed to support participants and reduce barriers to physical activity (23, 24) by providing (a) a free exercise manual with information, training programs (29), and video clips, (b) training equipment for coordination and strength, (c) travel cost reimbursement for study visits, and (d) weekly phone calls for support and motivation.

Participant feedback confirmed high utilization of materials and increased motivation through phone calls. High training compliance, adjusted intensity, and continued participation in half of the participants during the FU suggest that knowledge about the benefits of physical activity was effectively conveyed and implemented. As preferred by HNC patients (26), most trained alone, likely due to the individualized program.

The low-resource approach (patients: no travel time or costs, no costs for course participation or training equipment; therapists/clinics: no premises or costs for premises, no time commitment of therapists for the implementation of the training) appears suitable for routine aftercare but requires appropriately qualified exercise therapists for knowledge transfer.

### 4.4 Effects on QoL

The shown effect of individualized home training on the primary endpoint—global QoL—was lower than expected (30). Although the small intervention effect of our study with d=0.2023 is comparable to the findings of O’Neill et al. (50) this result is surprising, as our calculation for sample size acted on assumption of an experienced effect of d=0.5755, comparable to Burgos-Mansilla et al. (14).

The physical performance/functionality was not only objectively improved but also subjectively perceived by the participants. Strueder et al. (51) described a discrepancy between subjective QoL and objective functionality using swallowing function as an example. They attributed this difference, among other factors, to sensory deficits and reduced sensitivity, which can make it difficult to correctly perceive functional limitations.

In our cohort, global QoL increased by 8 points between the post-intervention (score=67) and FU assessments (score=75). In the literature, exact thresholds for clinically relevant differences are discussed with some controversy (52, 53). Although a threshold of ≥10 points is often assumed, it can be assumed that changes of 8 points are certainly clinically relevant, however small (52). While physical performance remained largely stable during this period, HNC-associated symptoms continued to decrease. These effects are less attributable to home training itself and more indicative of overall successful rehabilitative medical care. This suggests that global QoL is more strongly influenced by symptom reduction than by improvements in physical performance. Moreover, QoL is a multidimensional construct in which the physical component represents only one of several dimensions (54). Therefore, global QoL is not an adequate primary end point for assessing the effectiveness of exercise interventions (in HNC patients).

The improvement in emotional functioning corresponds to the subjective perception of mood enhancement. Whether this improvement is solely attributable to the training or rather the result of a multifactorial interaction—including symptom relief, increased social interactions, enhanced self-esteem, and improved sexual life—cannot be conclusively determined based on the available data. The reduction in dyspnea and, in particular, fatigue is likely due to the moderately intensive endurance training (43). However, the observed pain relief is primarily attributable to the intake of analgesics, which was reported by one-third of the participants, although exercise therapy may also contribute to pain reduction (46, 47).

### 4.5 Effects on physical activity level

The GSLTPAQ is a validated (34) and widely used tool in oncological research. Most studies utilize the LSI to categorize cancer survivors as either insufficiently active or active (35). In contrast, the present study focused on changes in activity level over time rather than categorial classification. Therefore, a modified version of the GSLTPAQ was used, consistent with approaches in many oncological studies (35). In addition to the LSI —calculated as the number of exercise sessions ≥15 minutes multiplied by their respective intensity levels —the average duration of these exercise sessions was recorded. The total weekly activity duration across the three measurement points showed only a slight increase during the intervention, from 280 to 290 minutes. However, the LSI rose significantly from 25 to 39, suggesting that participants performed their activities at higher intensities, consistent with our recommendations. This assumption is supported by the finding that the average training duration during the intervention (257 minutes per week) nearly matched the total weekly physical activity duration, indicating a shift toward more structured, higher-intensity exercise. Since higher training intensity is known to drive physiological adaptations, it is likely that the observed improvements in aerobic performance and muscle mass were primarily due to increase in intensity (49).

The findings—particularly the non-significant increase in LSI and the extended activity duration between post-intervention to FU—suggest a sustained enhancement in physical activity level, even though only about half of the participants continued with individual training. However, seasonal effects may have influenced the results: 40% of participants were enrolled in spring, leading to FU assessments in summer, a season associated with increased physical activity in the general population (55).

A small number of participants reported lower extremity pain following training, underscoring the importance of gradual training progression. Inactive or insufficiently active patients, in particular, should first increase training volume before advancing to higher intensities to reduce the risk of overuse injuries. For more accurate assessments of physical activity changes, future studies should consider using the modified GSLTPAQ or incorporate objective measurement tools.

### 4.6 Effects on body composition

During the intervention, positive changes in body composition were observed. The BMI remained stable, while muscle mass increased by approximately 3%p on average and fat mass decreased by around 1%p. The observed increase in muscle mass was only slightly lower than that reported following a progressive resistance training program (56). Since the evidence on the effects of exercise interventions on body composition in HNC patients during after care remains limited (16), a clear interpretation of the results is challenging. However, it is assumed that the positive effects can be attributed to the combination of individualized exercises, including strength exercise and endurance training. This training approach aligns with the current recommendations by Avancini et al. (16), who, based on existing evidence, consider a combination of strength and endurance training particularly effective for this patient population.

The lack of further muscle mass gain during the FU may possibly be attributed to the absence of progression in strength exercises. This highlights the importance of continuously adjusting training intensity and load to promote long-term muscular adaptations (49).

### 4.7 Effects on physical functioning

*Mobility:* Improvements in mobility across various body regions suggest the effectiveness of the individualized exercise program. Significant gains in mouth opening were observed after intervention, though additional therapies (e.g., physiotherapy, logopedics) were not tracked. At FU, no further significant gains in inter-incisor distance were observed, likely because most participants had already exceeded the functional threshold for trismus in HNC patients of 35 mm (57) or had reached a normal mouth opening of ≥40 mm (58). According to EORTC QLQ-HN35 assessments, fewer participants reported severe trismus symptoms at FU compared to pre-intervention.

Shoulder mobility improved in participants with restricted mobility in all three axes of movement, with mostly moderate to large effects. Head rotation improved in the home training group, as it did in the group training in the pilot cohort (28), but the effects were smaller in the former. In addition, improvements in ROM were observed in the home training group for lateral flexion of the cervical spine (moderate effect). It is assumed that in the home training group, the affected individuals trained more specifically than was possible in the group setting. Particularly in the cervical spine, ROM is not only determined by muscular tension but possibly also by blockages that cannot be addressed with self-training. Mobility of the lower back and hamstring (stand and reach test) improved markedly, with a large effect— greater than previously observed in group settings.

*Fall risk:* Although the SPPB has limited sensitivity in physical fit HNC patients (28), results suggest reduced fall risk through improved coordination and strength. The ceiling effect limited mensurable gains.

*Aerobic performance:* The 6MWT distance could be increased during the 12-week intervention despite the high baseline performance and was then maintained until the FU. The median distance of >580 m post-intervention and FU corresponds to the values of healthy subjects and is significantly higher than the distances that breast cancer survivors achieved on average (59, 60). The walking distance was almost identical to that of the pilot cohort (28) and comparable to the reports of Eades et al. (61) and Capozzi et al. (45). Walking was the most common form of endurance training in the study participants. The higher training intensity probably led to an increase in maximal oxygen uptake (38) as a result of the adaptation phenomena caused by stimuli that were now effective in training (49). The fact that the effect was large for the 6MWT is probably also due to the high specificity of the 6MWT.

*PROs:* The participants’ assessment of the extent of their improvements in the various areas is consistent with the objective measurement results. The improvement in mobility was most strongly perceived. The perceived improvement in coordination, which is mapped in functional diagnostics using the SPPB, appears stronger than the SPPB can map (lack of sensitivity). The less noticeable improvement in endurance performance was probably due to the adequacy of physical performance before the intervention. In addition, the improvement in walking distance (+31 m) was below the clinically significant change of 54 m for other clinical populations (62, 63). Consequently, the improvements in everyday activities were not as noticeable as the improvements in mobility. The same applies to the assessment of general performance. The improvement in strength was rated lowest by the participants. There were no objective measurement parameters for this, so a comparison was not possible. However, the strong improvements in mobility and the reduction in the risk of falling suggest that the participants focused on mobilization, coordination and stretching exercises. Consistent with the literature, regular training also had a positive effect on body awareness, mood and self-esteem (64, 65).

### 4.8 Additional comments on the assessment of medium-term sustainability

Overall, HNC patients are a vulnerable cohort, which is reflected by the many disease-related training interruptions and the high health-related dropout during the study (at least 8 of 53 corresponds to >15%). To avoid bias in the assessment of the sustainability of the training intervention, only patients who had completed the FU measurement (n=38) were included in this analysis. Consequently, the observed moderate to large intervention effects (pre-post analysis, n=44) faded out (**Table 2** *vs.* 4 and 3 *vs.* 5). This can be explained by the fact that the patients who completed the whole study (n=38) started with a better QoL (67 *vs.* 63, with (Q1 = 50, Q3 = 75) identical in both samples), lower symptom burden and better physical performance/functionality at the pre-intervention stage, than the cohort that only completed the pre-post measurements (n=44). Our results thus illustrate that the careful consideration of dropouts is important in longitudinal analyses, because dropouts can influence study results considerably, especially in small sized studies. Consequently, reported effects of exclusively short-term investigations should be interpreted at all times in its short-term context. And longer-term investigations should, at all measuring time points, include homogeneous samples. In future intervention studies, investigation ranges and study durations should be critically evaluated and discussed.

Since no significant changes were detectable between post- and FU measurements, one might conclude that observed short-term effects were maintained until the FU. However, it is to keep in mind, that in the case of a statistically significant short-term intervention effect with a subsequent medium-term effect (post-FU) of no significance, three true scenarios are possible here: a complete wash out of the short-term intervention effect (depending on time), a further increase, or, an equivalent situation. The letter two could be interpreted as sustainability of the obtained short-term effect and thus, a successful home-training intervention.

### 4.9 Safety

During the intervention, despite the high training volume, only a few adverse events occurred, none of which led to hospitalizations, long-term impairments, or damage. Consequently, in line with previous studies (44, 48, 61), home-based exercise training can be considered safe. However, it should be noted that HNC patients with clinically relevant comorbidities, including heart disease and severe chronic obstructive pulmonary disease, were excluded from study participation. To ensure safe training, the following recommendations are derived based on our approach: Firstly, exercise selection and intensity should be adapted to the existing limitations, comorbidities, and physical performance of the participants in accordance with current guidelines (43). Secondly, all exercises should be demonstrated and instructed by experienced therapists who can provide precise guidance on positioning and execution prior the training. Thirdly, for patients who are physically inactive or inexperienced, training volume and intensity should be increased gradually. We suspect that the reported pain, especially in the lower extremities (knees, Achilles tendon), is due to long endurance training sessions with sudden increases in intensity and could therefore be avoided.

### 4.10 Strengths and limitations

The OSHO#94 study ist he first study that evaluated the sustainability of a 100% home exercise program in addition to the effectiveness. Another strength is the high test specificity, particularly in assessing mobility and aerobic performance. However, the study design also has some limitations. In addition to the limitations already mentioned in the study protocol (30)— the single-arm design (lack of a control group), missing data on recruitment rates, and the use of the GSLTPAQ for assessing activity levels—the small sample size represents a relevant limitation. As a result, it was not possible to detect medium and small effects as statistically significant across three measurement time points, if there. And besides, we applied the Bonferroni correction as a conservative statistical method in multiple testing, knowing that our discernible set of study limitations makes conclusions much more difficult. Consequently, further well designed studies with a more appropriate primary outcome and larger sample sizes are needed to confirm our findings. Another limitation is the lack of an objective test for assessing muscle strength. A simple handgrip strength test could have provided additional information alongside changes in muscle mass. Despite these limitations, the results of the OSHO #94 trial demonstrate that an individualized home-based exercise program with low-to-moderate intensity is feasible, safe, and effective. The program, specifically designed for HNC patients, appears to be suitable for routine clinical care.

## 5 Conclusion and outlook

The individually adaptable home exercise program designed for HNC patients in aftercare proved to be safe and effective, particularly in terms of improving physical function. Additionally, positive changes were observed in body composition, along with an improvement in QoL. The high effectiveness was probably due to the consideration of three important training principles: The individualization/targeting of the training; progression, especially with regard to endurance training (increase in intensity); and the setting of effective training stimuli (49). Thus, the training program, including the chosen approach—knowledge transfer, free provision of training equipment, and remote support (for a certain period)— to be suitable for transferring the knowledge gained to date, primarily in randomized controlled trials, on the effectiveness of targeted exercise interventions in HNC patients (46, 47, 56, 66–70) into routine care. This could benefit HNC patients who have not previously had access to guided interventions, especially patients in rural areas. In addition, home training offers the advantage that the training can be adapted to the individual needs and preferences of the patients, is cost-effective and less time-consuming, which can potentially reduce barriers to participation in the training. Due to the specificity of functional diagnostics, particularly in mobility assessments and aerobic performance tests, even small effects could be detected. However, in our setting they could not be detected statistically significant. Studies with larger sample sizes and longer observation periods are necessary to determine whether less fit patients can achieve progress in their physical functioning beyond the supported phase.

## Data Availability

The datasets presented in this study can be found in online repositories. The names of the repository/repositories and accession number(s) can be found below: NFDI4Health repository: https://ldh.mediz-rostock.imise.uni-leipzig.de/projects/14.

## References

[1] Lo Nigro C Denaro N Merlotti A Merlano M. Head and neck cancer: improving outcomes with a multidisciplinary approach. Cancer Manag Res. (2017) 9:363–71. doi: 10.2147/CMAR.S115761 PMC5571817 28860859

[2] Ortiz-Comino L Fernández-Lao C Speksnijder CM Lozano-Lozano M Tovar-Martín I Arroyo-Morales M. Upper body motor function and swallowing impairments and its association in survivors of head and neck cancer: A cross-sectional study. PloS One. (2020) 15:e0234467. doi: 10.1371/journal.pone.0234467 32559241 PMC7304609

[3] Riechelmann H Dejaco D Steinbichler TB Lettenbichler-Haug A Anegg M Ganswindt U. Functional outcomes in head and neck cancer patients. Cancers (Basel). (2022) 14:1–19. doi: 10.3390/cancers14092135 PMC9099625 35565265

[4] van Hinte G Leijendekkers RA Merkx MAW Takes RP Nijhuis-van der Sanden MWG Speksnijder CM. Identifying unmet needs and limitations in physical health in survivors of Head and Neck Cancer. Eur J Cancer Care (Engl). (2021) 30:e13434. doi: 10.1111/ecc.13434 33709466 PMC8519003

[5] Baxi SS Schwitzer E Jones LW. A review of weight loss and sarcopenia in patients with head and neck cancer treated with chemoradiation. Cancers Head Neck. (2016) 1:5. doi: 10.1186/s41199-016-0010-0 31093339 PMC6460633

[6] Ellis MA Sterba KR Brennan EA Maurer S Hill EG Day TA. A systematic review of patient-reported outcome measures assessing body image disturbance in patients with head and neck cancer. Otolaryngol Head Neck Surg. (2019) 160:941–54. doi: 10.1177/0194599819829018 PMC6546516 30744514

[7] Epstein JB Robertson M Emerton S Phillips N Stevenson-Moore P. Quality of life and oral function in patients treated with radiation therapy for head and neck cancer. Head Neck. (2001) 23:389–98. doi: 10.1002/hed.v23:5 11295813

[8] Langendijk JA Doornaert P Verdonck-de Leeuw IM Leemans CR Aaronson NK Slotman BJ. Impact of late treatment-related toxicity on quality of life among patients with head and neck cancer treated with radiotherapy. J Clin Oncol. (2008) 26:3770–6. doi: 10.1200/JCO.2007.14.6647 18669465

[9] Chow LQM. Head and neck cancer. New Engl J Med. (2020) 382:60–72. doi: 10.1056/NEJMra1715715 31893516

[10] León X Orús C Casasayas M Neumann E Holgado A Quer M. Trends in disease-specific survival of head and neck squamous cell carcinoma patients treated in a single institution over a 30-year period. Oncol. (2021) 115:105184. doi: 10.1016/j.oraloncology.2021.105184 33581504

[11] Ferris RL Blumenschein G Fayette J Guigay J Colevas AD Licitra L. Nivolumab for recurrent squamous-cell carcinoma of the head and neck. N Engl J Med. (2016) 375:1856–67. doi: 10.1056/NEJMoa1602252 PMC5564292 27718784

[12] Ringash J Bernstein LJ Devins G Dunphy C Giuliani M Martino R. Head and neck cancer survivorship: learning the needs, meeting the needs. Semin Radiat Oncol. (2018) 28:64–74. doi: 10.1016/j.semradonc.2017.08.008 29173757

[13] Margalit DN Salz T Venchiarutti R Milley K McNamara M Chima S. Interventions for head and neck cancer survivors: Systematic review. Head Neck. (2022) 44:2579–99. doi: 10.1002/hed.v44.11 PMC9796901 35848095

[14] Burgos-Mansilla B Galiano-Castillo N Lozano-Lozano M Fernández-Lao C Lopez-Garzon M Arroyo-Morales M. Effect of physical therapy modalities on quality of life of head and neck cancer survivors: A systematic review with meta-analysis. J Clin Med. (2021) 10:1–18. doi: 10.3390/jcm10204696 PMC8539984 34682818

[15] Pérez IMM Pérez SEM García RP Lupgens D Martínez GB González CR. Exercise-based rehabilitation on functionality and quality of life in head and neck cancer survivors. A systematic review and meta-analysis. Sci Rep. (2023) 13:8523. doi: 10.1038/s41598-023-35503-y 37237097 PMC10219996

[16] Avancini A Borsati A Belluomini L Giannarelli D Nocini R Insolda J. Effect of exercise across the head and neck cancer continuum: a systematic review of randomized controlled trials. Support Care Cancer. (2023) 31:670. doi: 10.1007/s00520-023-08126-2 37924500 PMC10625510

[17] Bye A Sandmael JA Stene GB Thorsen L Balstad TR Solheim TS. Exercise and nutrition interventions in patients with head and neck cancer during curative treatment: A systematic review and meta-analysis. Nutrients. (2020) 12:1–24. doi: 10.3390/nu12113233 PMC7690392 33105699

[18] Capozzi LC Nishimura KC McNeely ML Lau H Culos-Reed SN. The impact of physical activity on health-related fitness and quality of life for patients with head and neck cancer: a systematic review. Br J Sports Med. (2016) 50:325–38. doi: 10.1136/bjsports-2015-094684 25966911

[19] Goyal N Day A Epstein J Goodman J Graboyes E Jalisi S. Head and neck cancer survivorship consensus statement from the American Head and Neck Society. Laryngoscope Invest Otolaryngol. (2022) 7:70–92. doi: 10.1002/lio2.v7.1 PMC8823162 35155786

[20] Fang Y-Y Wang C-P Chen Y-J Lou P-J Ko J-Y Lin J-J. Physical activity and fitness in survivors of head and neck cancer. Support Care Cancer. (2021) 29:6807–17. doi: 10.1007/s00520-021-06192-y 33997941

[21] Karczewska-Lindinger M Tuomi L Fridolfsson J Arvidsson D Börjesson M Finizia C. Low physical activity in patients diagnosed with head and neck cancer. Laryngoscope Invest Otolaryngol. (2021) 6:747–55. doi: 10.1002/lio2.v6.4 PMC8356879 34401499

[22] Felser S Grosse-Thie C Rogahn J Koch T Strueder D Busch CJ. Accelerometer based physical activity in patients with head and neck cancer. Jahrestagung der DGEpi: DGEpi, University Ulm (2022) p. 298, Abstract Book 17.

[23] Ning Y Wang Q Ding Y Zhao W Jia Z Wang B. Barriers and facilitators to physical activity participation in patients with head and neck cancer: a scoping review. Support Care Cancer. (2022) 30:4591–601. doi: 10.1007/s00520-022-06812-1 35032199

[24] Doughty HC Hill RA Riley A Midgley AW Patterson JM Boddy LM. Barriers to and facilitators of physical activity in adults living with and beyond cancer, with special emphasis on head and neck cancer: a systematic review of qualitative and mixed methods studies. Support Care Cancer. (2023) 31:471. doi: 10.1007/s00520-023-07925-x 37458858 PMC10352410

[25] Midgley AW Lowe D Levy AR Mepani V Rogers SN. Exercise program design considerations for head and neck cancer survivors. Eur Arch oto-rhino-laryngology. (2018) 275:169–79. doi: 10.1007/s00405-017-4760-z PMC5754417 29058083

[26] Rogers LQ Malone J Rao K Courneya KS Fogleman A Tippey A. Exercise preferences among patients with head and neck cancer: prevalence and associations with quality of life, symptom severity, depression, and rural residence. Head Neck. (2009) 31:994–1005. doi: 10.1002/hed.21053 19340875

[27] Jackson C Dowd AJ Capozzi LC Bridel W Lau HY Culos-Reed SN. A turning point: Head and neck cancer patients’ exercise preferences and barriers before and after participation in an exercise intervention. Eur J Cancer Care (Engl). (2018) 27:e12826. doi: 10.1111/ecc.2018.27.issue-2 29377317

[28] Felser S Behrens M Strüder D Liese J Rohde K Junghanss C. Feasibility and effects of a supervised exercise program suitable for independent training at home on physical function and quality of life in head and neck cancer patients: A pilot study. Integr Cancer Ther. (2020) 19:1–12. doi: 10.1177/1534735420918935 PMC7265079 32476513

[29] (2019). Kiefer-, Gesichts- und Halstumoren. Rostock: Übungshandbuch für Patienten mit Mund-.

[30] Felser S Rogahn J Glass Ä Bonke LA Strüder DF Stolle J. Feasibility of individualized home exercise programs for patients with head and neck cancer–study protocol and first results of a multicentre single-arm intervention trial (OSHO 94). PloS One. (2024) 19:1–18. doi: 10.1371/journal.pone.0301304 PMC11341025 39173016

[31] Borg GA. Psychophysical bases of perceived exertion. Med Sci Sports Exerc. (1982) 14:377–81. doi: 10.1249/00005768-198205000-00012 7154893

[32] Aaronson NK Ahmedzai S Bergman B Bullinger M Cull A Duez NJ. The European Organization for Research and Treatment of Cancer QLQ-C30: a quality-of-life instrument for use in international clinical trials in oncology. J Natl Cancer Inst. (1993) 85:365–76. doi: 10.1093/jnci/85.5.365 8433390

[33] Bjordal K de Graeff A Fayers PM Hammerlid E van Pottelsberghe C Curran D. A 12 country field study of the EORTC QLQ-C30 (version 3.0) and the head and neck cancer specific module (EORTC QLQ-H&N35) in head and neck patients. Eur J Cancer. (2000) 36:1796–807. doi: 10.1016/S0959-8049(00)00186-6 10974628

[34] Amireault S Godin G. The Godin-Shephard leisure-time physical activity questionnaire: validity evidence supporting its use for classifying healthy adults into active and insufficiently active categories. Percept Mot Skills. (2015) 120:604–22. doi: 10.2466/03.27.PMS.120v19x7 25799030

[35] Amireault S Godin G Lacombe J Sabiston CM. The use of the Godin-Shephard Leisure-Time Physical Activity Questionnaire in oncology research: a systematic review. BMC Med Res Methodol. (2015) 15:60. doi: 10.1186/s12874-015-0045-7 26264621 PMC4542103

[36] Holt LE Pelham TW Burke DG. Modifications to the standard sit-and-reach flexibility protocol. J Athl Train. (1999) 34:43–7. PMC1322873 16558547

[37] Guralnik JM Ferrucci L Pieper CF Leveille SG Markides KS Ostir GV. Lower extremity function and subsequent disability: consistency across studies, predictive models, and value of gait speed alone compared with the short physical performance battery. J Gerontol A Biol Sci Med Sci. (2000) 55:M221–31. doi: 10.1093/gerona/55.4.M221 PMC12149745 10811152

[38] Schmidt K Vogt L Thiel C Jäger E Banzer W. Validity of the six-minute walk test in cancer patients. Int J Sports Med. (2013) 34:631–6. doi: 10.1055/s-0032-1323746 23444095

[39] Borg E Borg G Larsson K Letzter M Sundblad B-M. An index for breathlessness and leg fatigue. Scand J Med Sci Sports. (2010) 20:644–50. doi: 10.1111/j.1600-0838.2009.00985.x 19602182

[40] Sullivan GM Feinn R. Using effect size-or why the P value is not enough. J Grad Med Educ. (2012) 4:279–82. doi: 10.4300/JGME-D-12-00156.1 PMC3444174 23997866

[41] Cohen J. Statistical power analysis for the behavioral sciences. 2. Hillsdale, NJ: Erlbaum (1988).

[42] Midgley AW Levy AR Price R Cunha FA Rogers SN. Should survivors of head and neck cancer be considered a distinct special population within the context of exercise prescription? Br J Maxillofac Surg. (2020) 58:738–43. doi: 10.1016/j.bjoms.2020.03.021 32624268

[43] Campbell KL Winters-Stone KM Wiskemann J May AM Schwartz AL Courneya KS. Exercise guidelines for cancer survivors: consensus statement from international multidisciplinary roundtable. Med Sci Sports Exerc. (2019) 51:2375–90. doi: 10.1249/MSS.0000000000002116 PMC8576825 31626055

[44] Cnossen IC van Uden-Kraan CF Rinkel RNPM Aalders IJ de Goede CJT de Bree R. Multimodal guided self-help exercise program to prevent speech, swallowing, and shoulder problems among head and neck cancer patients: a feasibility study. J Med Internet Res. (2014) 16:e74. doi: 10.2196/jmir.2990 24610383 PMC3961811

[45] Capozzi LC Boldt KR Lau H Shirt L Bultz B Culos-Reed SN. A clinic-supported group exercise program for head and neck cancer survivors: managing cancer and treatment side effects to improve quality of life. Sports Med. (2015) 23:1001–7. doi: 10.1007/s00520-014-2436-4 25256377

[46] McNeely ML Parliament M Courneya KS Seikaly H Jha N Scrimger R. A pilot study of a randomized controlled trial to evaluate the effects of progressive resistance exercise training on shoulder dysfunction caused by spinal accessory neurapraxia/neurectomy in head and neck cancer survivors. Head Neck. (2004) 26:518–30. doi: 10.1002/hed.20010 15162353

[47] McNeely ML Parliament MB Seikaly H Jha N Magee DJ Haykowsky MJ. Effect of exercise on upper extremity pain and dysfunction in head and neck cancer survivors: a randomized controlled trial. Cancer. (2008) 113:214–22. doi: 10.1002/cncr.v113:1 18457329

[48] Kok A Passchier E May AM van den Brekel MWM Jager-Wittenaar H Veenhof C. Feasibility of a supervised and home-based tailored exercise intervention in head and neck cancer patients during chemoradiotherapy. Eur J Cancer Care (Engl). (2022) 31:e13662. doi: 10.1111/ecc.13662 35953883 PMC9787293

[49] Ferrauti A ed. Trainingswissenschaft für die Sportpraxis: Lehrbuch für Studium, Ausbildung und Unterricht im Sport. Berlin, Heidelberg: Springer Spektrum (2020). doi: 10.1007/978-3-662-58227-5

[50] O’Neill LM Guinan E Doyle SL Bennett AE Murphy C Elliott JA. The RESTORE randomized controlled trial: impact of a multidisciplinary rehabilitative program on cardiorespiratory fitness in esophagogastric cancer survivorship. Ann Surg. (2018) 268:747–55. doi: 10.1097/SLA.0000000000002895 30004915

[51] Strüder D Ebert J Kalle F Schraven SP Eichhorst L Mlynski R. Head and neck cancer: A study on the complex relationship between qoL and swallowing function. Curr Oncol. (2023) 30:10336–50. doi: 10.3390/curroncol30120753 PMC10742452 38132387

[52] Cocks K King MT Velikova G Fayers PM Brown JM. Quality, interpretation and presentation of European Organisation for Research and Treatment of Cancer quality of life questionnaire core 30 data in randomised controlled trials. Eur J Cancer. (2008) 44:1793–8. doi: 10.1016/j.ejca.2008.05.008 18599286

[53] Mouelhi Y Jouve E Castelli C Gentile S. How is the minimal clinically important difference established in health-related quality of life instruments? Review of anchors and methods. Health Qual Life Outcomes. (2020) 18:136. doi: 10.1186/s12955-020-01344-w 32398083 PMC7218583

[54] World Health Organization Quality of Life. What quality of life? The WHOQOL Group. World Health Organization Quality of Life Assessment. World Health Forum. (1996) 17:354–6. 9060228

[55] Shephard RJ Aoyagi Y. Seasonal variations in physical activity and implications for human health. Eur J Appl Physiol. (2009) 107:251–71. doi: 10.1007/s00421-009-1127-1 19609553

[56] Lønbro S Dalgas U Primdahl H Johansen J Nielsen JL Aagaard P. Progressive resistance training rebuilds lean body mass in head and neck cancer patients after radiotherapy–results from the randomized DAHANCA 25B trial. Radiother Oncol. (2013) 108:314–9. doi: 10.1016/j.radonc.2013.07.002 23932192

[57] Dijkstra PU Huisman PM Roodenburg JLN. Criteria for trismus in head and neck oncology. Int J Maxillofac Surg. (2006) 35:337–42. doi: 10.1016/j.ijom.2005.08.001 16280237

[58] Deutsche Gesellschaft für Funktionsdiagnostik und -therapie (DGFDT). CMD-screening (CMD-basisdiagnostik) (2024). Available online at: https://www.dgfdt.de/documents/266840/22655647/CMD-Screening+DGFDT+2024/ac5abfe4-604d-4c84-9851-e426a54edc91 (Accessed March 5, 2025).

[59] Moser N. Körperliche Leistungsfähigkeit gemessen anhand der Sechs-Minuten-Gehstrecke in der Normalbevölkerung – Determinanten, Referenzperzentile und Zusammenhang mit der selbstberichteten körperlichen Leistungsfähigkeit und Gesundheit in der populationsbasierten STAAB Kohortenstudie. Würzburg: Inauguraldissertation: Julius-Maximilians-Universität Würzburg (2022). doi: 10.25972/OPUS-32958

[60] But-Hadzic J Dervisevic M Karpljuk D Videmsek M Dervisevic E Paravlic A. Six-minute walk distance in breast cancer survivors-A systematic review with meta-analysis. Int J Environ Res Public Health. (2021) 18:1–13. doi: 10.3390/ijerph18052591 PMC7967367 33807611

[61] Eades M Murphy J Carney S Amdouni S Lemoignan J Jelowicki M. Effect of an interdisciplinary rehabilitation program on quality of life in patients with head and neck cancer: review of clinical experience. Head Neck. (2013) 35:343–9. doi: 10.1002/hed.22972 22422558

[62] Redelmeier DA Bayoumi AM Goldstein RS Guyatt GH. Interpreting small differences in functional status: the Six Minute Walk test in chronic lung disease patients. Am J Respir Crit Care Med. (1997) 155:1278–82. doi: 10.1164/ajrccm.155.4.9105067 9105067

[63] Wise RA Brown CD. Minimal clinically important differences in the six-minute walk test and the incremental shuttle walking test. COPD. (2005) 2:125–9. doi: 10.1081/COPD-200050527 17136972

[64] Tikac G Unal A Altug F. Regular exercise improves the levels of self-efficacy, self-esteem and body awareness of young adults. J Sports Med Phys Fitness. (2022) 62:157–61. doi: 10.23736/S0022-4707.21.12143-7 33555673

[65] Mikkelsen K Stojanovska L Polenakovic M Bosevski M Apostolopoulos V. Exercise and mental health. Maturitas. (2017) 106:48–56. doi: 10.1016/j.maturitas.2017.09.003 29150166

[66] Capozzi LC McNeely ML Lau HY Reimer RA Giese-Davis J Fung TS. Patient-reported outcomes, body composition, and nutrition status in patients with head and neck cancer: Results from an exploratory randomized controlled exercise trial. Cancer. (2016) 122:1185–200. doi: 10.1002/cncr.v122.8 26828426

[67] Hong Y-L Hsieh T-C Chen P-R Chang S-C. Nurse-led counseling intervention of postoperative home-based exercise training improves shoulder pain, shoulder disability, and quality of life in newly diagnosed head and neck cancer patients. J Clin Med. (2022) 11:1–12. doi: 10.3390/jcm11144032 PMC9315873 35887795

[68] McGarvey AC Hoffman GR Osmotherly PG Chiarelli PE. Maximizing shoulder function after accessory nerve injury and neck dissection surgery: A multicenter randomized controlled trial. Head Neck. (2015) 37:1022–31. doi: 10.1002/hed.23712 25042422

[69] Su T-L Chen A-N Leong C-P Huang Y-C Chiang C-W Chen I-H. The effect of home-based program and outpatient physical therapy in patients with head and neck cancer: A randomized, controlled trial. Oncol. (2017) 74:130–4. doi: 10.1016/j.oraloncology.2017.10.002 29103741

[70] Thomas A D’Silva C Mohandas L Pais SMJ Samuel SR. Effect of muscle energy techniques V/S active range of motion exercises on shoulder function post modified radical neck dissection in patients with head and neck cancer - A randomized clinical trial. Asian Pac J Cancer Prev. (2020) 21:2389–93. doi: 10.31557/APJCP.2020.21.8.2389 PMC7771923 32856870

