# Supplementary material for: Individualized home training in head and neck cancer patients is safe and has positive short- and medium-term effects –results of a multicenter, single-arm intervention trial (OSHO #94)
Source: Front Oncol. 2025 Jun 9;15:1602532. doi: 10.3389/fonc.2025.1602532 (PMC12183252; doi:10.3389/fonc.2025.1602532)
Supplement: Supplementary file 1 [file DataSheet1.pdf]

Figure S1. Recommended and completed training

A

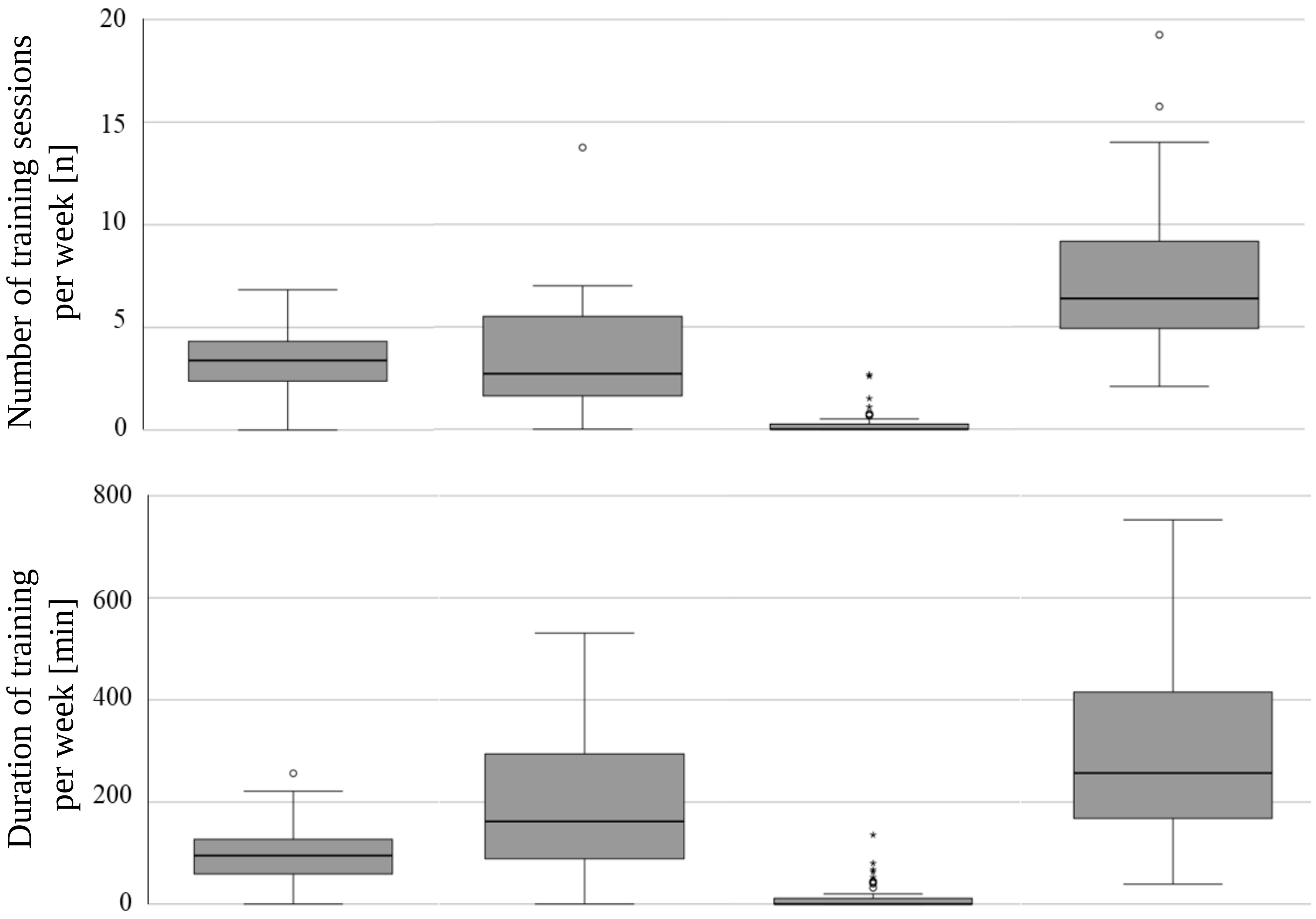

B

Weekly…	Individual n = 41	Endurance n = 40	Additional n = 41	**Total n = 40**
… recommended training sessions [n]	≥3	2 to 3	-	**≥5**
… completed training sessions [n]	3.3 (2.2, 4.3)	2.7 (1.6, 5.5)	0.0 (0.0, 0.3)	**6.4 (4.9, 9.2)**
… recommended training duration [min]	≥45	60 to 90	-	**≥105**
… completed training duration [min]	95 (56, 127)	162 (86, 304)	0 (0, 11)	**257 (162, 417)**
**C** Recommendations followed [n (%)]	35 (85)	31 (78)	-	**28 (68)**

Part A displays the distribution of the documented average weekly training sessions and durations completed by participants, separately for individual training, endurance training, non-recommended activities (additional), and total training sessions/duration.
Part B juxtaposes the recommended training sessions and durations with the actual training performed (median (Q1, Q3)).
Part C shows the proportion of participants who met the recommendations for individual training, endurance training, and both categories (total).
