# Supplementary material for: Individualized home training in head and neck cancer patients is safe and has positive short- and medium-term effects –results of a multicenter, single-arm intervention trial (OSHO #94)
Source: Front Oncol. 2025 Jun 9;15:1602532. doi: 10.3389/fonc.2025.1602532 (PMC12183252; doi:10.3389/fonc.2025.1602532)
Supplement: Supplementary file 2 [file DataSheet2.pdf]

Individualized home training in head and neck cancer patients is safe and has positive short- and medium-term effects – results of a multicenter, single-arm intervention trial (OSHO #94)

Figure S2. Extent of subjective improvements (n = 43)

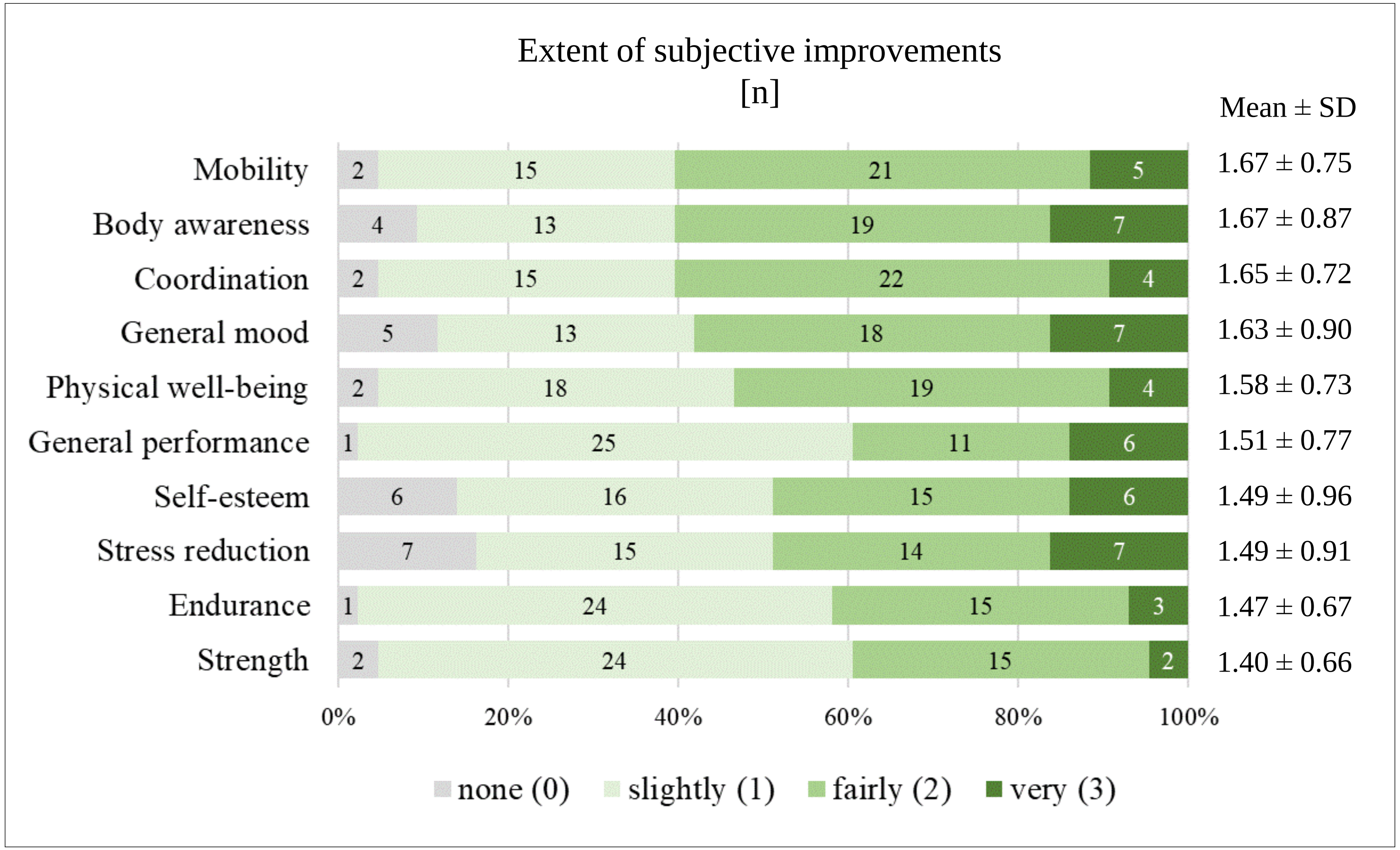

Participants (n=43) rated after the 12-week intervention the perceived effects on ten domains using a 4-point Likert scale from 0=none to 3=very. Domains are ordered by magnitude (mean), from highest (top) to lowest (bottom).
