# Supplementary material for: Individualized home training in head and neck cancer patients is safe and has positive short- and medium-term effects –results of a multicenter, single-arm intervention trial (OSHO #94)
Source: Front Oncol. 2025 Jun 9;15:1602532. doi: 10.3389/fonc.2025.1602532 (PMC12183252; doi:10.3389/fonc.2025.1602532)

Individualized home training in head and neck cancer patients is safe and has positive short- and medium-term effects –results of a multicenter, single-arm intervention trial (OSHO #94)

Figure S3. Distribution of quality of life parameters assessed by participants using the EORTC QLQ-C30 and EORTC QLQ-HN35 questionnaires, as well as parameters related to physical activity measured using the Godin-Shephard Leisure-Time Physical Activity Questionnaire, across the three time points: pre-intervention, post-intervention, and follow-up (n = 38)

Quality of life - EORTC QLQ-C30

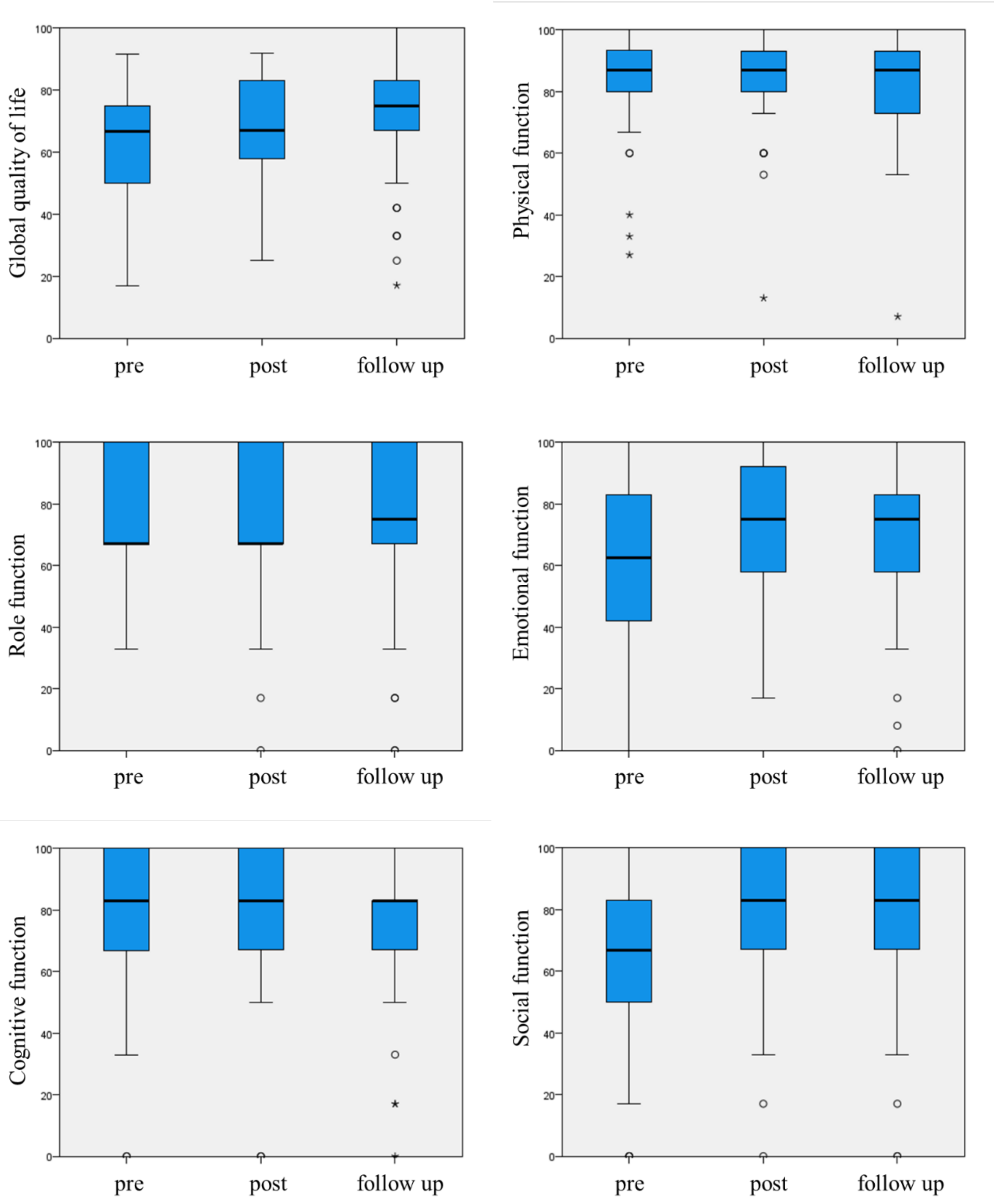

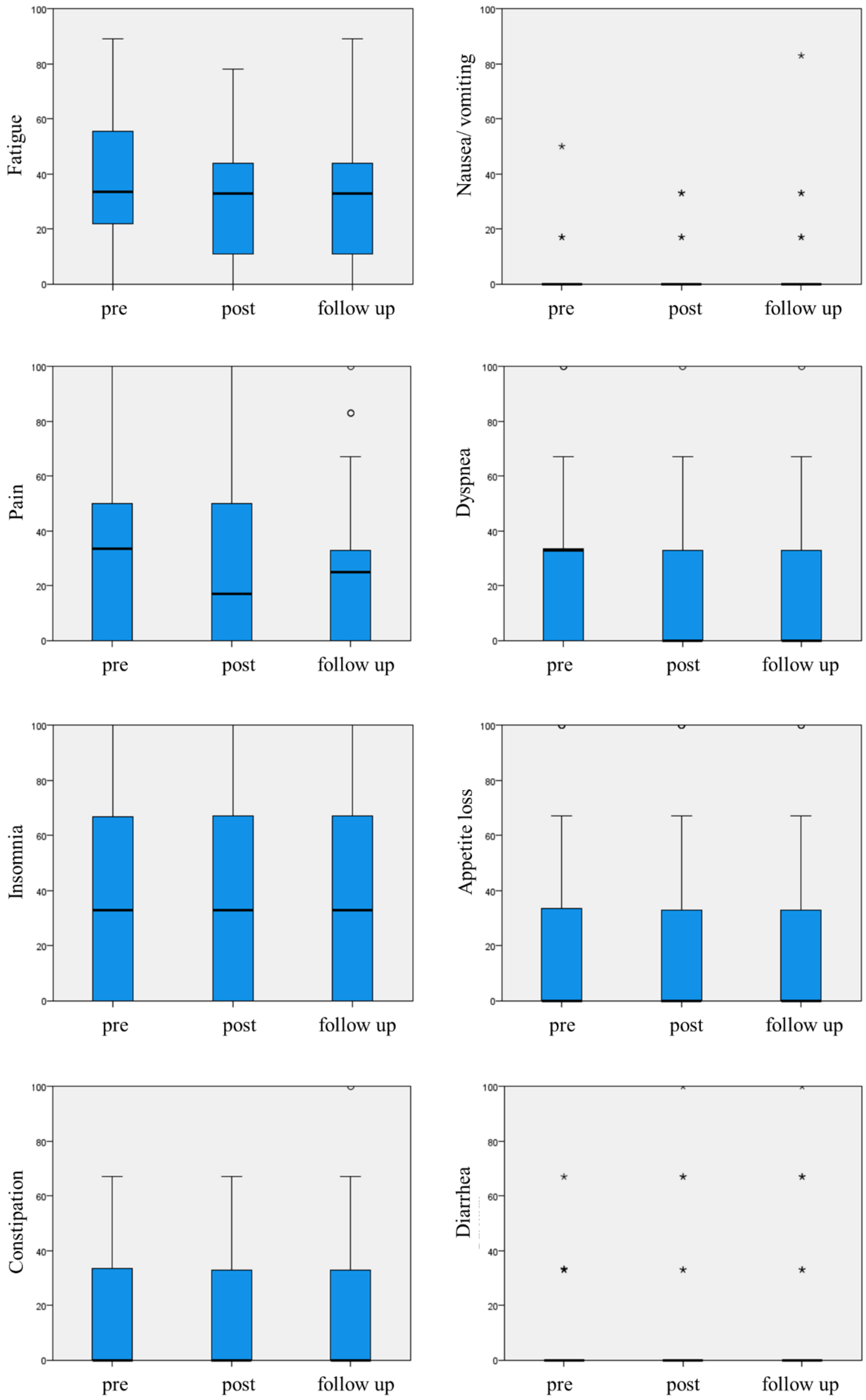
Fatigue
Nausea/ vomiting
Pain
Dyspnea
Insomnia
Appetite loss
Constipation
Diarrhea
pre
post
follow up
0
20
40
60
80
100

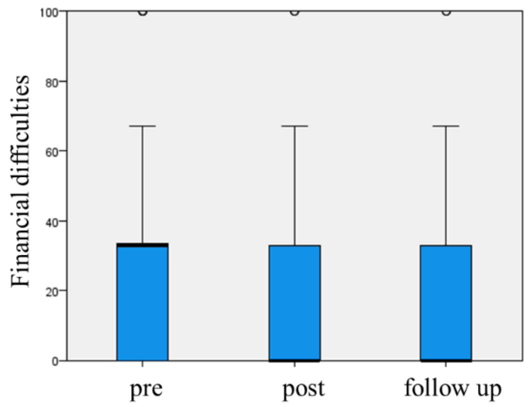

Quality of life - EORTC QLQ-HN35

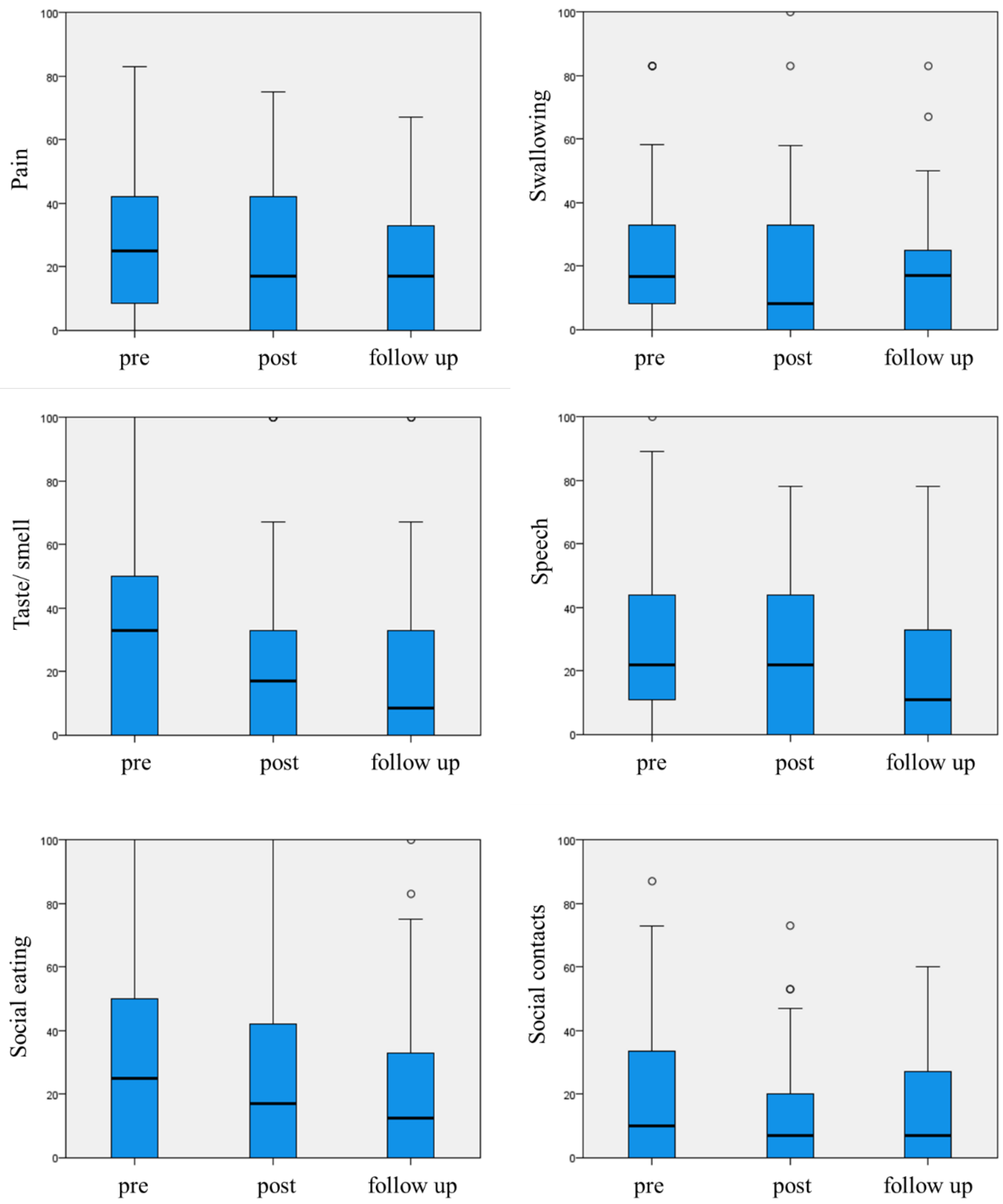

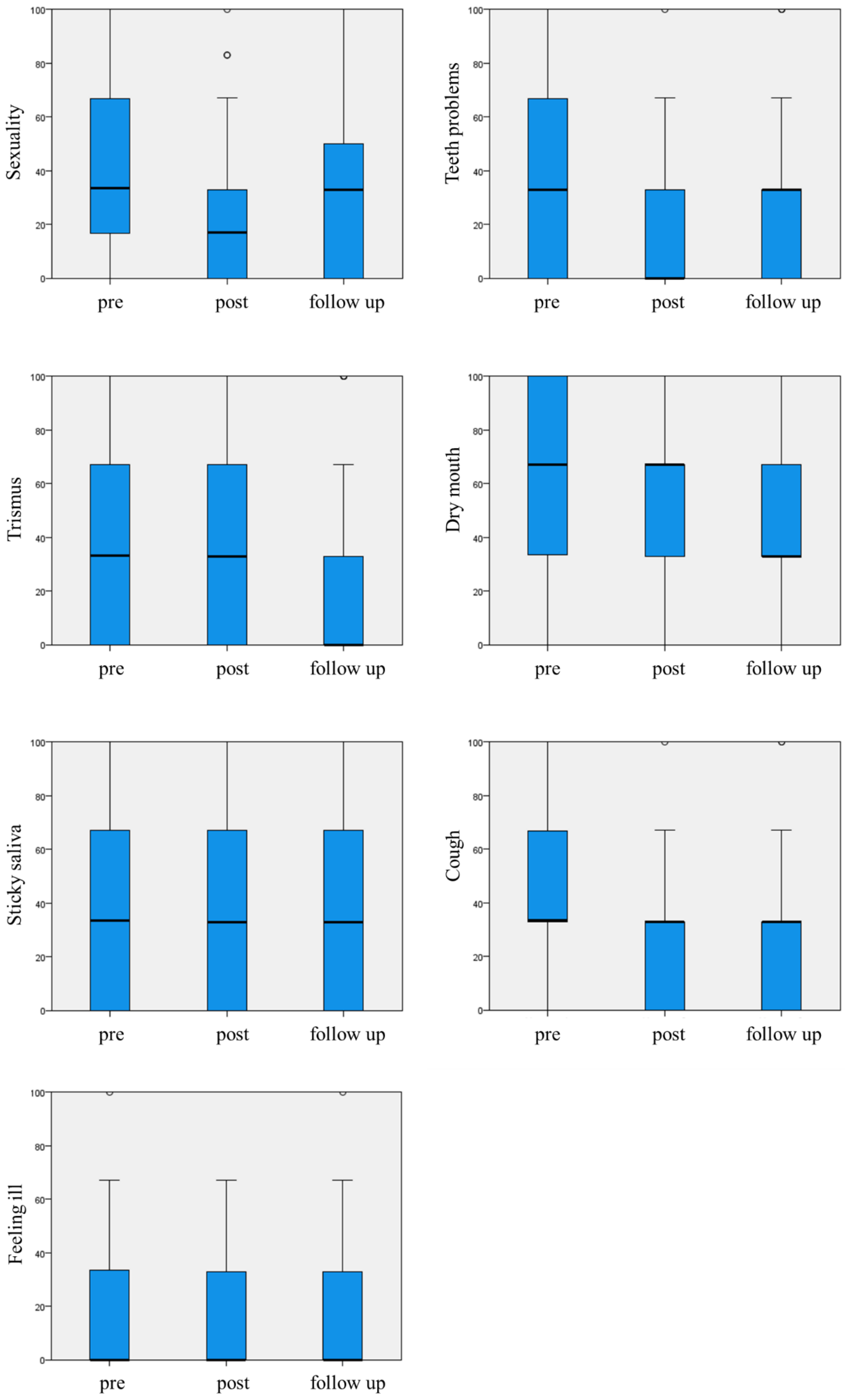

Sexuality
Teeth problems
Trismus
Dry mouth
Sticky saliva
Cough
Feeling ill
0
20
40
60
80
100
pre
post
follow up

Physical activity - Godin-Shepard Leisure-Time Physical Activity Questionnaire

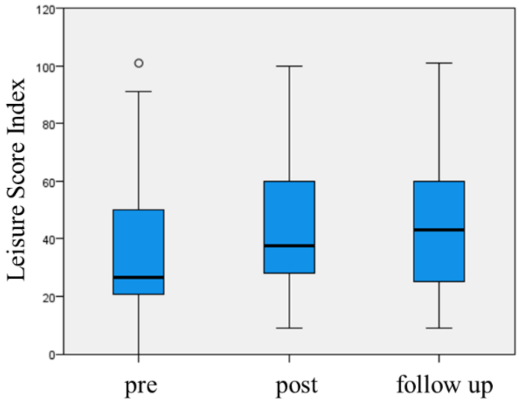

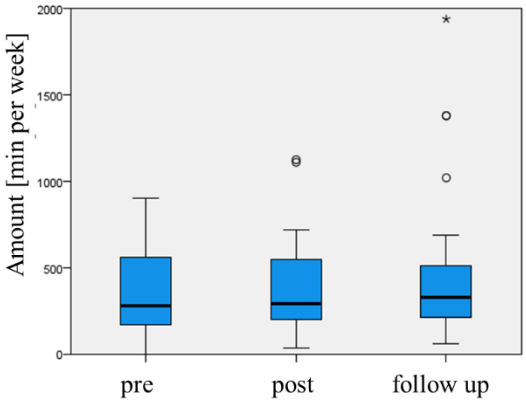

Supplement: Supplementary file 3 [file DataSheet3.pdf]
