# Supplementary material for: Individualized home training in head and neck cancer patients is safe and has positive short- and medium-term effects –results of a multicenter, single-arm intervention trial (OSHO #94)
Source: Front Oncol. 2025 Jun 9;15:1602532. doi: 10.3389/fonc.2025.1602532 (PMC12183252; doi:10.3389/fonc.2025.1602532)

Individualized home training in head and neck cancer patients is safe and has positive short- and medium-term effects – results of a multicenter, single-arm intervention trial (OSHO #94)

Figure S4. Distribution of parameters related to body composition and physical functionality, assessed by participants at the three time points: pre-intervention, post-intervention, and follow-up

Body composition

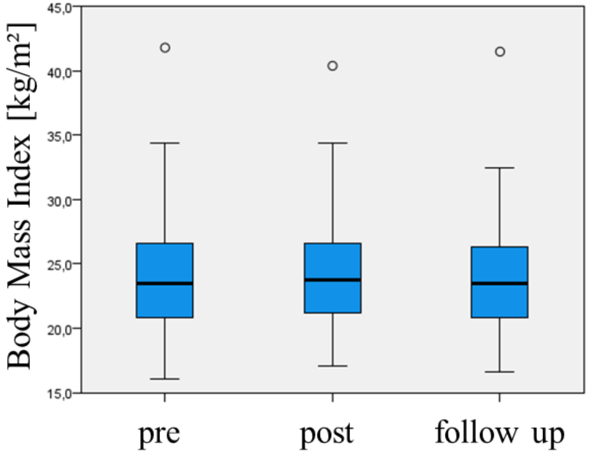

(n=31)

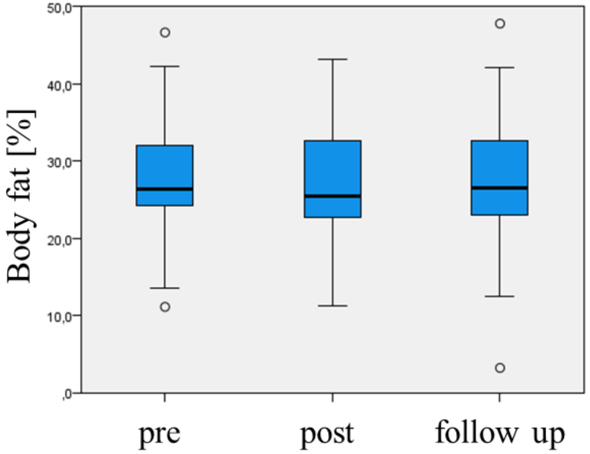

(n=29)

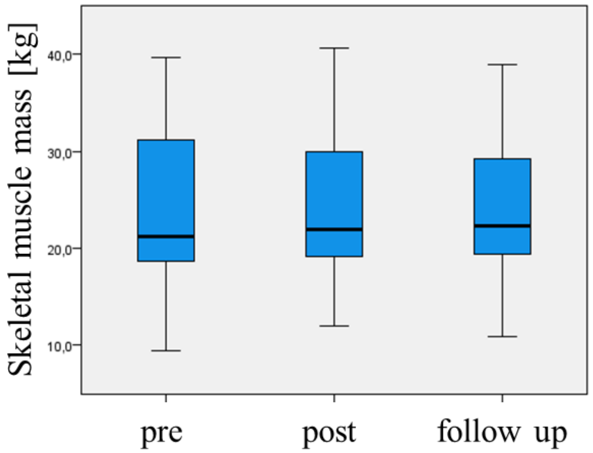

(n=29)

Flexibility of the temporomandibular joints (n=27)

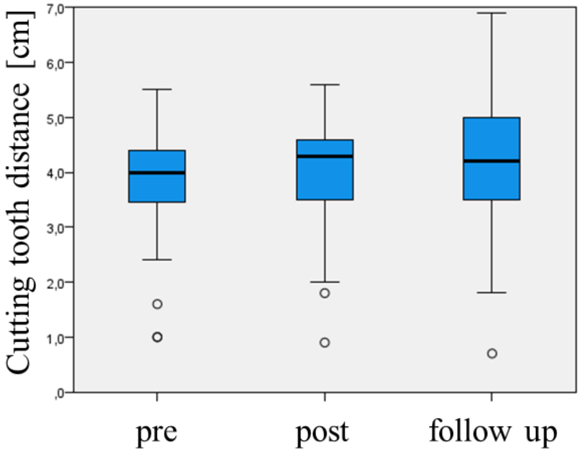

Flexibility of the shoulder joints

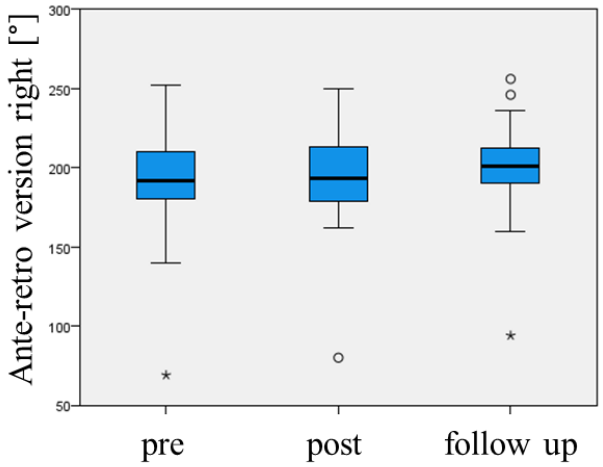

(n=32)

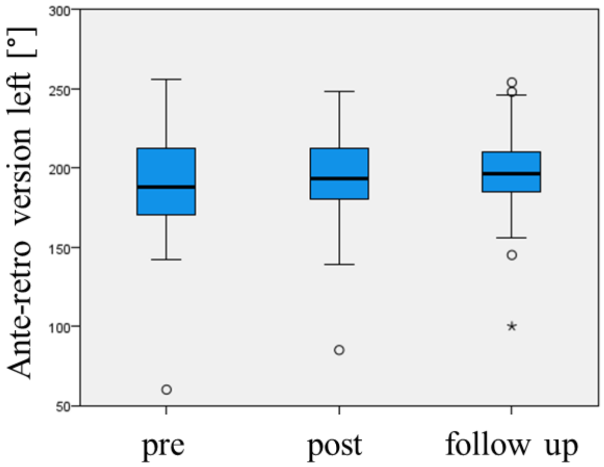

(n=31)

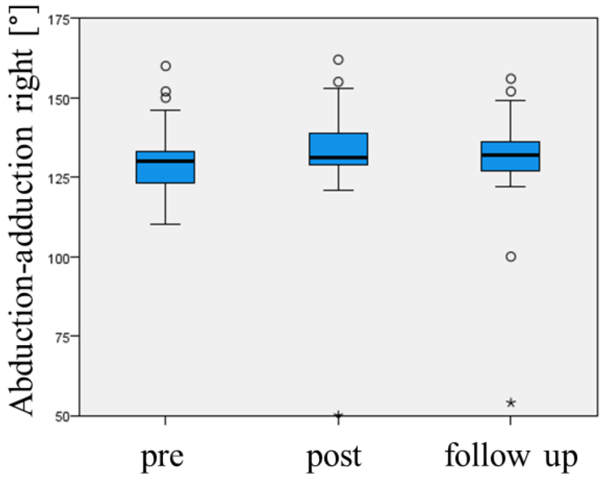

(n=33)

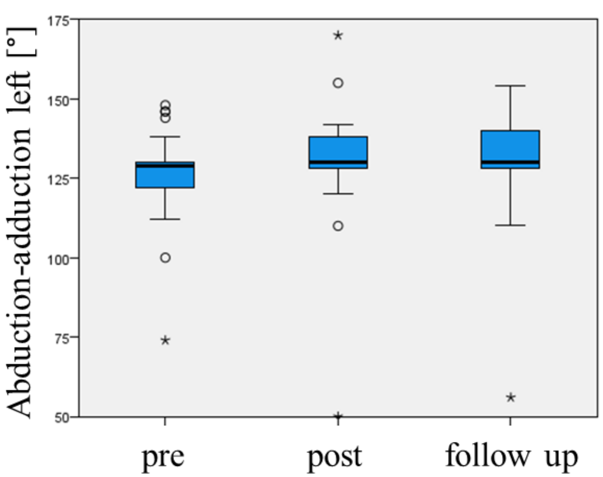

(n=34)

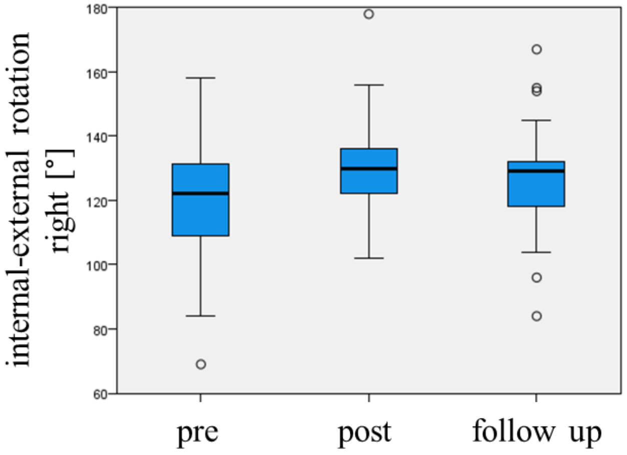

(n=35)

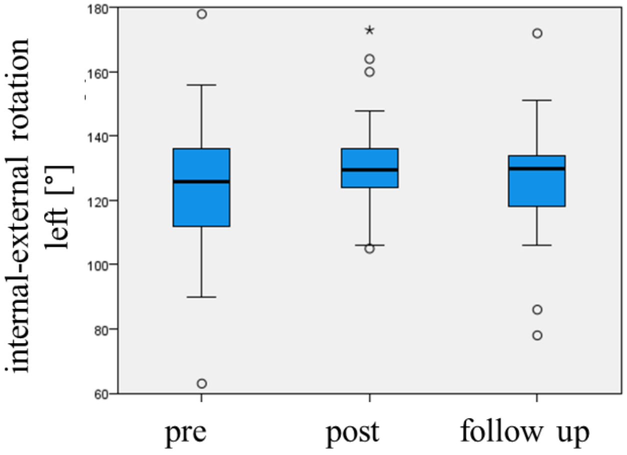

(n=33)

Flexibility of the cervical spine

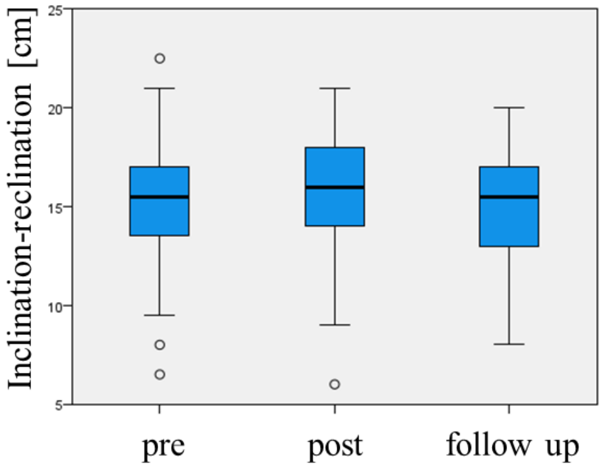

(n=37)

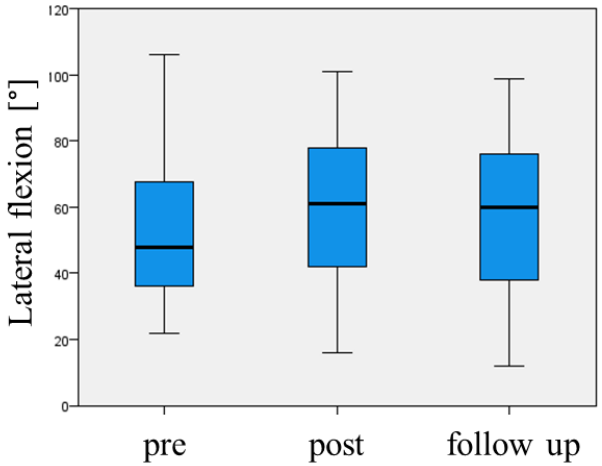

(n=35)

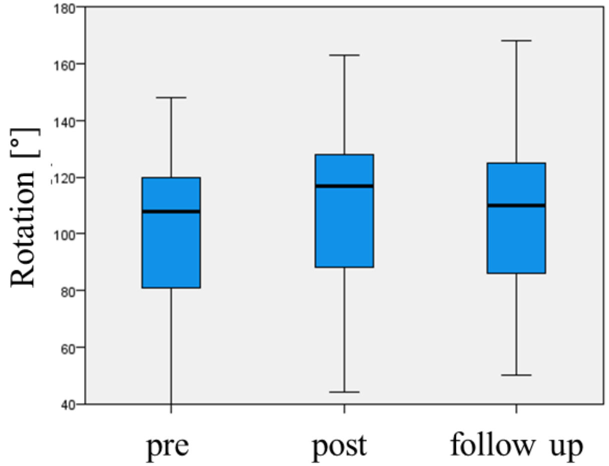

(n=37)

Flexibility of the lower back and hamstring muscles (n=34)

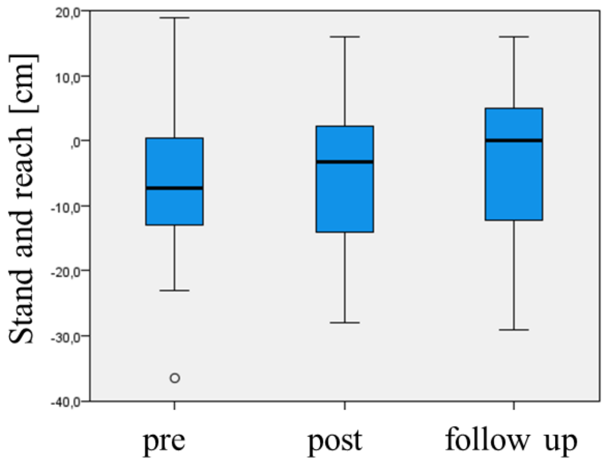

Fall risk (n=35)

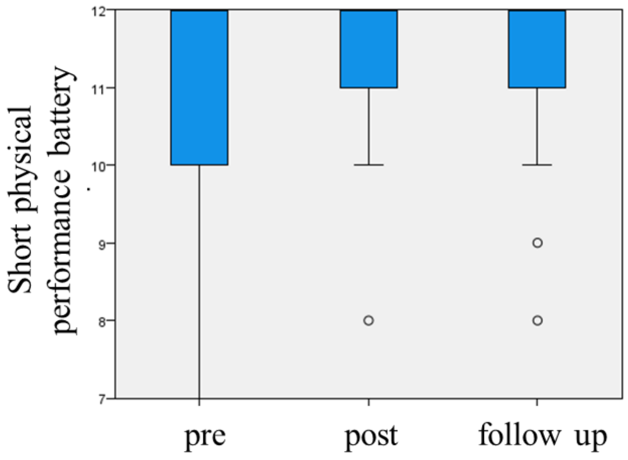

Aerobic performance (n=31)

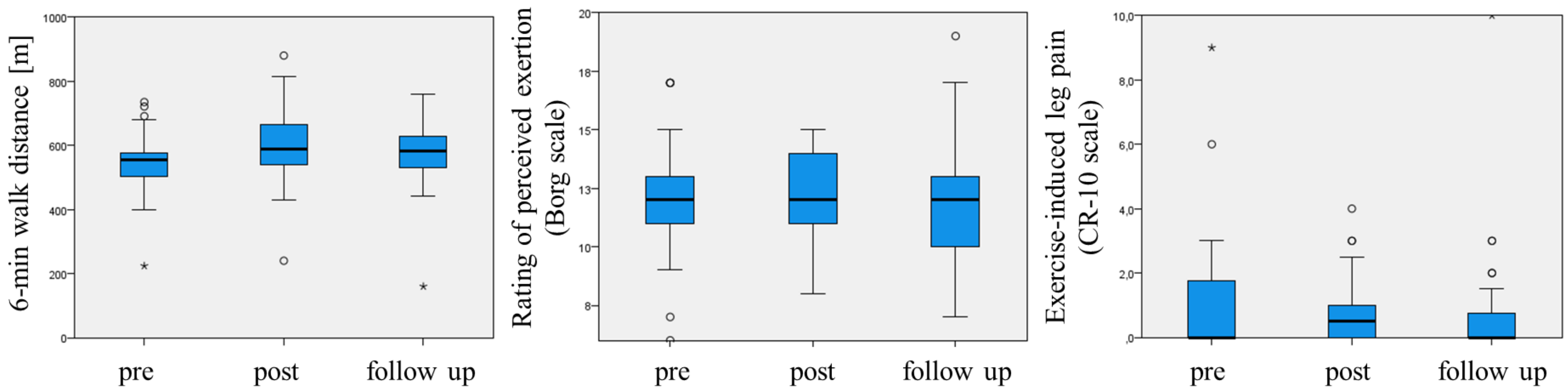

Supplement: Supplementary file 4 [file DataSheet4.pdf]
